# Your verbal questions beginning with '*what*' will rapidly deactivate the left prefrontal cortex of listeners

**DOI:** 10.1038/s41598-021-84610-1

**Published:** 2021-03-04

**Authors:** Hirotaka Iwaki, Masaki Sonoda, Shin-ichiro Osawa, Brian H. Silverstein, Takumi Mitsuhashi, Kazushi Ukishiro, Yutaro Takayama, Toshimune Kambara, Kazuo Kakinuma, Kyoko Suzuki, Teiji Tominaga, Nobukazu Nakasato, Masaki Iwasaki, Eishi Asano

**Affiliations:** 1 Department of Pediatrics, Children’s Hospital of Michigan, Wayne State University, Detroit, MI 48201 USA; 2 Department of Neurology, Children’s Hospital of Michigan, Wayne State University, Detroit, MI 48201 USA; 3 Department of Epileptology, Tohoku University Graduate School of Medicine, Sendai, 9808575 Japan; 4 Department of Neurosurgery, Tohoku University Graduate School of Medicine, Sendai, 9808575 Japan; 5 Department of Behavioral Neurology and Cognitive Neuroscience, Tohoku University Graduate School of Medicine, Sendai, 9808575 Japan; 6 Department of Neurosurgery, Graduate School of Medicine, Yokohama City University, Kanagawa, 2360004 Japan; 7 Department of Neurosurgery, National Center of Neurology and Psychiatry, National Center Hospital, Tokyo, 1878551 Japan; 8 Department of Psychology, Hiroshima University, Hiroshima, 7398524 Japan; 9 Translational Neuroscience Program, Wayne State University, Detroit, MI 48201 USA; 10 Department of Neurosurgery, School of Medicine, Juntendo University, Tokyo, 1138421 Japan

**Keywords:** Epilepsy, Language

## Abstract

The left prefrontal cortex is essential for verbal communication. It remains uncertain *at what timing, to what extent, and what type of phrase* initiates left-hemispheric dominant prefrontal activation during comprehension of spoken sentences. We clarified this issue by measuring event-related high-gamma activity during a task to respond to three-phrase questions configured in different orders. Questions beginning with a *wh-*interrogative *deactivated* the left posterior prefrontal cortex right after the 1st phrase offset and the anterior prefrontal cortex after the 2nd phrase offset. Left prefrontal high-gamma activity augmented subsequently and maximized around the 3rd phrase offset. Conversely, questions starting with a concrete phrase deactivated the right orbitofrontal region and then *activated* the left posterior prefrontal cortex after the 1st phrase offset. Regardless of sentence types, high-gamma activity emerged earlier, by one phrase, in the left posterior prefrontal than anterior prefrontal region. Sentences beginning with a *wh-*interrogative may initially deactivate the left prefrontal cortex to prioritize the bottom-up processing of upcoming auditory information. A concrete phrase may obliterate the inhibitory function of the right orbitofrontal region and facilitate top-down lexical prediction by the left prefrontal cortex. The left anterior prefrontal regions may be recruited for semantic integration of multiple concrete phrases.

## Introduction

**Figure 1:**
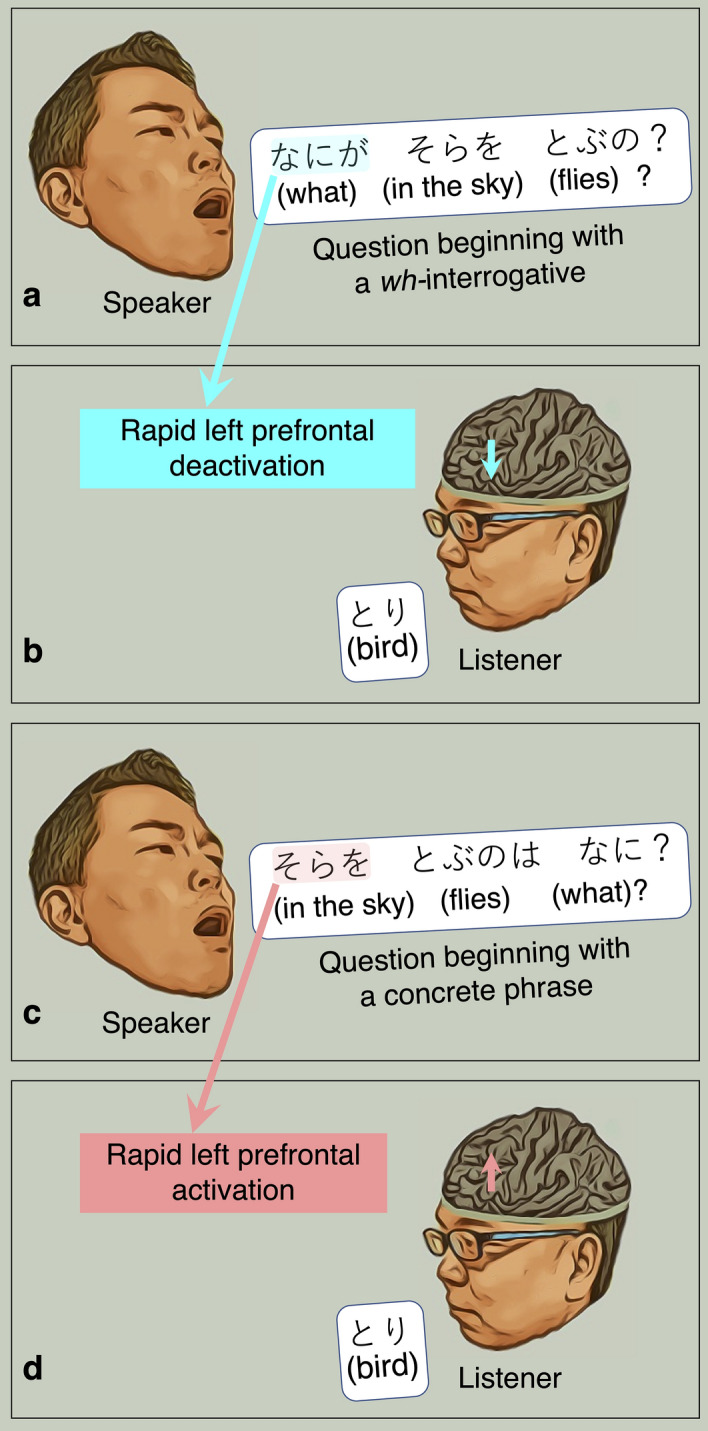
Four-frame cartoon summarizing the results of the present study. (**a**) and (**b**) The present study will demonstrate that verbal questions beginning with a *wh-*interrogative *deactivate* the left posterior prefrontal regions immediately after the 1st phrase offset. (**c**) and (**d**) Conversely, those beginning with a concrete word *activate* the left posterior prefrontal regions immediately after the 1st phrase offset. It should be noted that an adposition follows a noun in Japanese but precedes in English.

This study will demonstrate that a verbal question beginning with a *wh-*interrogative (*what*, *when*, or *where*) rapidly deactivates the left prefrontal cortex of listeners (Fig. 1). This statement may surprise some of the readers because it appears to contradict the general belief that spoken language is processed predominantly in the left cerebral hemisphere in adolescents and adults^1–4^. Studies using lesion-deficit analysis^5^, intracarotid amobarbital test^6^, and electrical stimulation mapping^7^ have indicated that the left perisylvian regions are essential to comprehend spoken sentences and to generate speech outputs. The left superior- and middle-temporal gyri (STG and MTG) are suggested to decode acoustic inputs into phonemes and words in a *bottom-up* manner^1–4^. Electrical stimulation of the left STG and MTG often induces a transient inability to construct a lexical item from speech sounds^7,8^. The left prefrontal regions within the inferior- and middle-frontal gyri (IFG and MFG) are suggested to be involved in the prediction and integration of the lexical items in a *top-down* manner as well as in the determination of the semantic context expressed by a spoken sentence^1–4^. Electrical stimulation of the left IFG and MFG frequently induces a temporary inability to form verbal answers to auditory sentence questions with the ability to replicate a vocal sound being maintained^8,9^.

Studies using functional MRI (fMRI) and intracranial EEG (iEEG) likewise have suggested that the left perisylvian regions play predominant roles in the processing of spoken language. Tasks requiring sentence comprehension were reported to elicit hemodynamic and neuronal activation in the left perisylvian areas more intensely than in the right homotopic areas^6,8,10–12^. Right-hemispheric language dominance is a rare observation unless a given patient is left-handed and suffers from a congenital epileptogenic lesion involving the left neocortical areas^13–15^. Though many investigators have studied language-related neuronal activation heavily, it remains to be addressed *at what timing, to what extent, and what type of phrase* initiates left-hemispheric dominant prefrontal activation during comprehension of spoken sentences.

**Figure 2:**
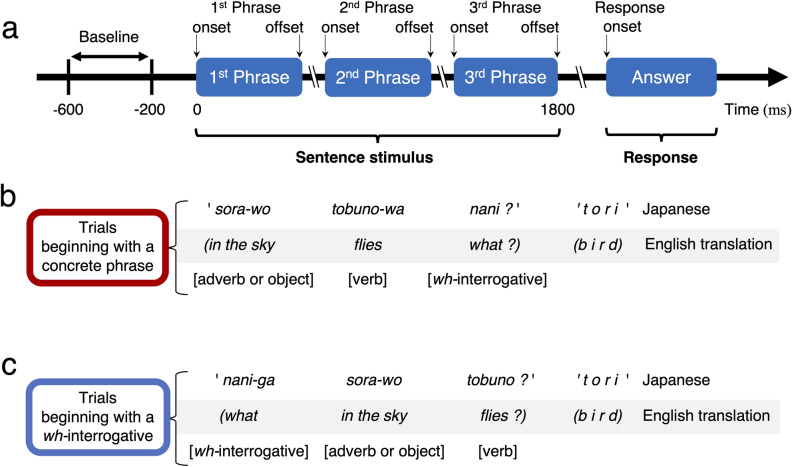
Spoken sentence stimuli used for measurement of task-related high-gamma modulations. (**a**) Patients were instructed to listen to a series of sentence questions and overtly provide a relevant answer to each item during extraoperative intracranial EEG recording. The response time was defined as the period between sentence offset (i.e., 3rd phrase offset) and response onset. A 400-ms resting period, at 200–600 ms before the stimulus onset, was treated as the baseline period for measurement of task-related high-gamma modulations. We have prepared 192 sentence stimuli, in total, for the present study. (**b**) Ninety-six stimuli were characterized by the following order of three phrases: [adverb or object], [verb], and [what, when, or where]. Each of the 96 sentence stimuli begins with a concrete word; [adverb or object] starts with a concrete noun because an adposition (e.g., ’-o’) follows a noun in Japanese but precedes in English (e.g., ’in the’). (**c**) Each of the other 96 sentence stimuli (each with the same semantic context as one of the former 96) begins with a wh-interrogative and is characterized by the following order of three phrases: [what, when, or where], [adverb or object], and [verb]. A given patient was assigned 48 question stimuli beginning with a concrete phrase and 48 beginning with a wh-interrogative; thereby, none of the assigned 96 questions shared the same semantic context. Sentence types were counterbalanced across patients.

We will address this question and improve our understanding of the neurobiology of language by investigating native Japanese-speaking patients undergoing extraoperative iEEG recordings as a part of the presurgical epilepsy evaluation. In Japanese, the phrase order is flexible, a sentence question with the same meaning can begin with a *wh-*interrogative or concrete phrase, and native speakers commonly use both phrase orders (Fig. 2)^16^. The present study will test the three hypotheses, as mentioned below. (i) We hypothesize that left prefrontal activation and right prefrontal deactivation will take place at the 1st phrase offset for the lexical processing. Our hypothesis is in part based on the following notion. During language processes, each hemisphere is suggested to interact with the other^17^; thereby, the right prefrontal cortex is indicated to exert an inhibitory control^18–22^. Thus, we expect that deactivation of the right prefrontal cortex will precede the left prefrontal activation. Deactivation of the inhibitory function of the right prefrontal cortex is expected to effectively facilitate the lexical prediction process by the left prefrontal region. (ii) We hypothesize that the magnitude of left prefrontal activation at the 1st phrase offset will be higher when a sentence question begins with a concrete phrase compared to a *wh-*interrogative. This hypothesis is based on our expectation that a concrete word would provide listeners with semantic contexts while a *wh-*interrogative per se would not. (iii) We hypothesize that a single concrete phrase will trigger left posterior prefrontal cortical activation, whereas the accumulation of multiple concrete phrases will trigger left anterior prefrontal activation. This hypothesis is primarily driven by the observations of prior fMRI studies that increased semantic loads were associated with increased hemodynamic activation in the left anterior prefrontal region, including the Brodmann Area 47 (BA47)^23^.

We will maximize the generalizability of our iEEG observations using mixed model analysis, which controls the effects of patient demographics, focal epilepsy, and antiepileptic drugs on task-related neuronal modulations. The present study has quantified the degree of task-related neuronal modulation of an underlying cortex using event-related high-gamma activity^8,24^. The magnitudes of high-gamma modulations are tightly correlated to neuronal firing on a single-cell recording^25^, hemodynamic modulations of fMRI^26,27^, and glucose metabolism on positron emission tomography^28^. The signal fidelity of iEEG is > 100 times better than that of scalp recording^29^. Resection of sites showing language task-related high-gamma augmentation frequently results in a postoperative language impairment^30^. In contrast, the clinical significance of resecting sites showing event-related modulations of lower frequency band activities is less understood.

## Results

### Behavioral observations

Twenty-three patients satisfied the inclusion and exclusion criteria (age range: 11–54 years; 10 females; Table 1). A total of 1,119 artifact-free non-epileptic electrodes (i.e., outside the seizure onset zone, interictal spiking zone, and structural lesions^31,32^) were available for further analysis (626: left hemisphere and 493: right hemisphere; Table 2).

**Table 1:** Patient profiles.

Characteristics	
Median age (years) [SD]	27.5 [10.8]
Median age at epilepsy onset (years) [SD]	13.8 [11.2]
Female, n (%)	10 (43.5)
**Seizure onset zone, n (%)**	
Left frontal	4 (17.4)
Left temporal	7 (30.4)
Left parieto-occipital	1 (4.3)
Right frontal	3 (13.0)
Right temporal	4 (17.4)
Right parieto-occipital	4 (17.4)
**Sampled hemisphere, n**	
Left	11
Right	9
Left and right	3
Median number of antiepileptic drugs [SD]	3.17 [1.05]
Median FIQ [SD]	81.7 [14.2]
**Etiology, n (%)**	
Tumor	3 (13.0)
Focal dysplasia	11 (47.8)
No lesion other than gliosis	3 (13.0)
Hippocampal sclerosis	2 (8.7)
Focal ulegyria	2 (8.7)
Suspected encephalitis	1 (4.3)
Arteriovenous malformation	1 (4.3)

*SD* standard deviation, *FIQ* full-scale intelligence quotient.

**Table 2:** The number of electrodes at regions of interest (ROIs).

ROIs	Left	Right
Posterior middle-frontal gyrus (MFG)	31 (4)	18 (4)
Anterior middle-frontal gyrus	38 (7)	23 (4)
Posterior inferior-frontal gyrus (IFG; BA44/45)	30 (7)	20 (5)
Orbitofrontal region (BA47 and BA12)*	22 (6)	18 (4)
Supramarginal gyrus	57 (10)	34 (8)
Inferior precentral gyrus	27 (6)	30 (7)
Posterior superior-temporal gyrus (STG)	49 (9)	26 (6)
Posterior middle-temporal gyrus (MTG)	24 (7)	15 (7)
Others	348 (14)	309 (12)
Total	626 (14)	493 (12)

The total number of analyzed electrode sites (and the number of contributing patients) in each ROI is provided. The Desikan FreeSurfer Atlas was used to define ROIs in the present study^8,66^.

*BA* Brodmann area.

*The orbitofrontal region was defined as the summation of the pars orbitalis of the IFG and the lateral orbitofrontal gyrus.

The response time did not differ between trials beginning with a concrete phrase and those starting with a *wh-*interrogative (median across 23 patients: 1,488 ms vs. 1,695 ms; *p* = 0.390 on Wilcoxon Signed Rank Test; Supplementary Table S1). Likewise, the proportion of correct answers did not differ between the two sentence types mentioned above (median: 97.9% vs. 97.9%; *p* = 0.682).

### Visualization of the dynamics of neuronal modulations supporting sentence comprehension

**Figure 3:**
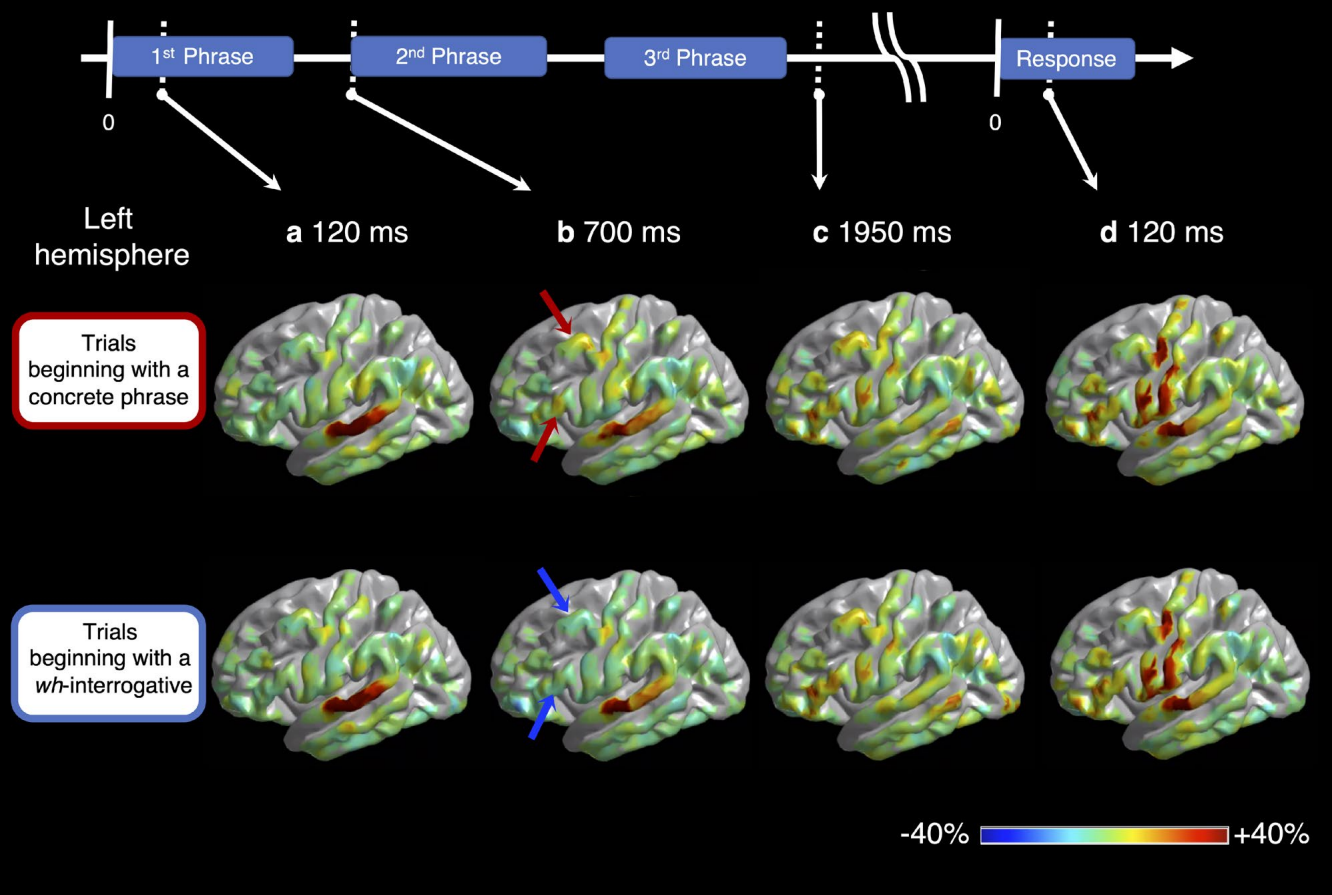
Snapshots of task-related high-gamma modulations. The video snapshots demonstrate the percent change of high-gamma activity relative to the baseline period (i.e., between − 600 and − 200 ms relative to stimulus onset). (**a**) At 120 ms following the 1st phrase onset, the bilateral superior-temporal gyri (STG) showed high-gamma augmentation. (**b**) At 700 ms following stimulus onset (i.e., after the 1st phrase offset), high-gamma activity in the left posterior prefrontal region was augmented during trials beginning with a concrete phrase (red arrow) but suppressed during those beginning with a wh-interrogative (blue arrow). (**c**) At 1,950 ms following the 1st phrase onset (i.e., after the 3rd phrase offset), high-gamma augmentation involved the left temporal and frontal lobe regions, extensively, commonly across the sentence types. (**d**) At 120 ms after response onset, high-gamma augmentation involved the Rolandic areas and STG bilaterally.

Supplementary Videos S1 and S2 demonstrate the spatiotemporal dynamics of cortical activation and deactivation during the sentence comprehension task, as reflected by high-gamma augmentation and suppression. High-gamma augmentation initially took place at the bilateral STG during sentence listening (Fig. 3a), gradually involved extensive areas of the left temporal and frontal regions (Fig. 3b,c) and finally involved the bilateral Rolandic regions during overt responses (Fig. 3d). We have provided the statistical results in detail below.

### Left prefrontal high-gamma modulations during comprehension of a sentence beginning with a *wh-*interrogative

**Figure 4:**
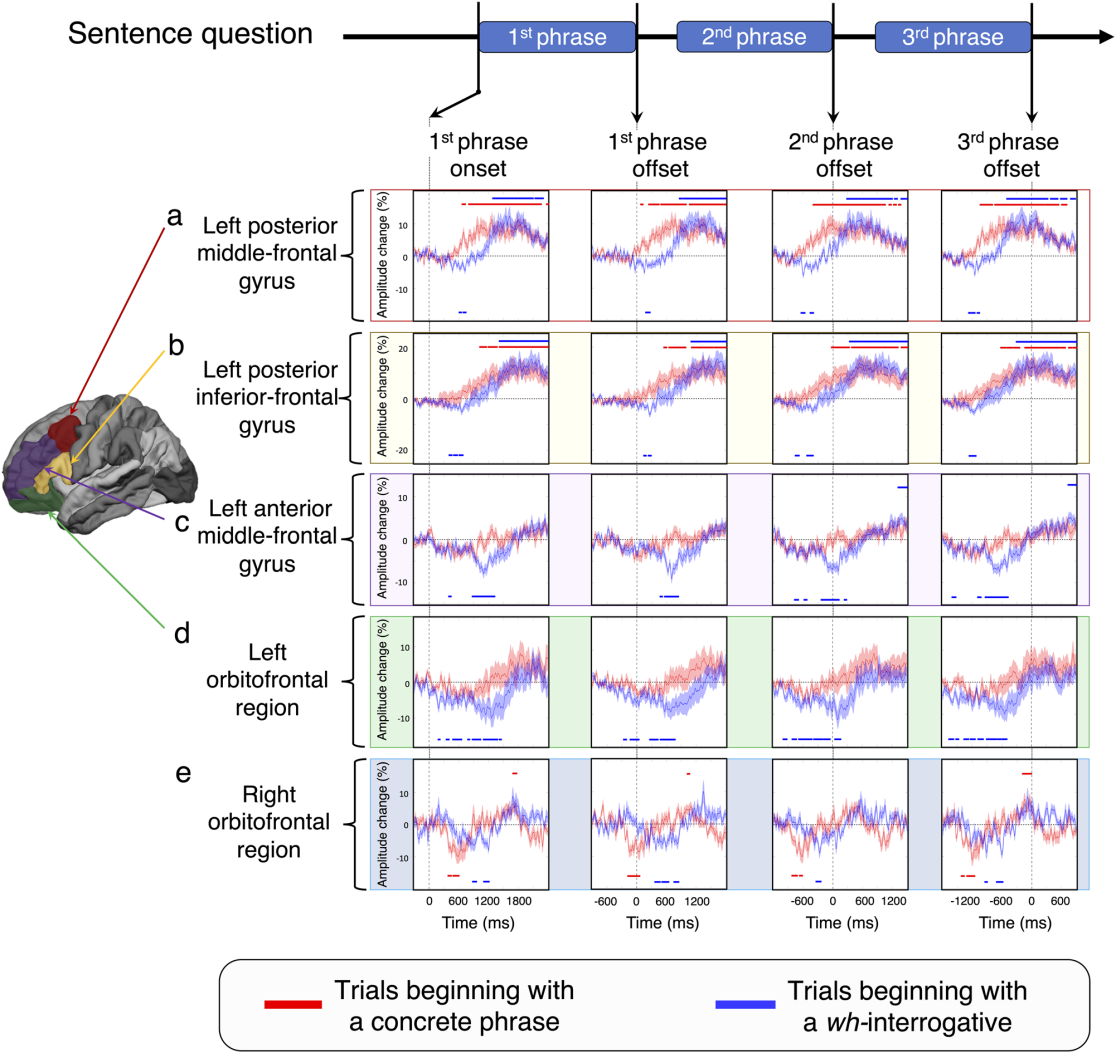
The dynamics of high-gamma modulations during sentence comprehension task. (**a**)-(**d**) High-gamma modulations in the left hemisphere. (**e**) High-gamma modulations in the right hemisphere. The mean percent change in high-gamma activity is presented with a standard error bar. Red plots and bars: Trials beginning with a concrete phrase. Blue plots and bars: Trials beginning with a *wh*-interrogative. Upper horizontal bars: Significant amplitude augmentation lasting at least 60 ms (including at least four 65 Hz-band oscillations). Lower horizontal bars: Significant amplitude suppression lasting at least 60 ms. First column: Time-locked to the 1st phrase onset (i.e., sentence onset). Second column: Time-locked to the 1st phrase offset. Third column: Time-locked to the 2nd phrase offset. Fourth column: Time-locked to the 3rd phrase offset (i.e., sentence offset). (**a**) and (**b**) The left posterior middle- and inferior-frontal gyri (MFG and IFG) showed a rising of high-gamma activity around the 1st phrase offset during trials beginning with a concrete phrase. These regions showed high-gamma suppression maximally around the 1st phrase offset during trials beginning with a *wh-*interrogative. (**c**) and (**d**) The left anterior MFG and orbitofrontal regions showed high-gamma suppression maximally around the 2nd phrase offset during trials beginning with a *wh-*interrogative. (**e**) The right orbitofrontal region showed high-gamma suppression maximally before the 1st phrase offset during trials beginning with a concrete phrase.

Questions beginning with a *wh-*interrogative rapidly *deactivated* the left posterior prefrontal regions, and such prefrontal high-gamma suppression took place in a posterior-to-anterior direction. The magnitude of high-gamma suppression at the left posterior MFG reached the maximum at 150 ms after the 1st phrase offset (Fig. 4a; maximum suppression =  − 5.3%; 95% Confidence Interval [CI]: − 7.0 to − 3.7%; false discovery rate [FDR]-adjusted *p* = 0.006). Likewise, the magnitude of high-gamma suppression at the left posterior IFG was maximized at 260 ms after the 1st phrase offset (Fig. 4b; maximum suppression =  − 6.2%; 95% CI − 8.3 to − 4.2%; FDR-adjusted *p* = 0.006). The maximum high-gamma suppression at the left anterior MFG (Fig. 4c; maximum suppression =  − 7.2%; 95% CI − 9.6 to − 4.9%; FDR-adjusted *p* = 0.007) and left orbitofrontal region (Fig. 4d; maximum suppression =  − 11.5%; 95% CI − 16.7 to − 5.4%; FDR-adjusted *p* = 0.010) took place at 110 ms and 110 ms after the 2nd phrase offset, respectively.

Left prefrontal high-gamma activity subsequently rose and maximized around or after the 3rd phrase offset (Fig. 4). The left posterior prefrontal high-gamma activity reached the maximum earlier than the left anterior. Specifically, the magnitude of high-gamma augmentation at the left posterior MFG reached the statistical significance at 500 ms before the 3rd phrase offset and the maximum at 190 ms before the 3rd phrase offset (Fig. 4a; maximum augmentation =  + 12.9%; 95%CI + 8.5 to + 18.2%; FDR-adjusted *p* = 0.005). Likewise, high-gamma augmentation at the left posterior IFG reached the statistical significance at 310 ms before the 3rd phrase offset and the maximum at 310 ms after the 3rd phrase offset (Fig. 4b; maximum augmentation =  + 15.1%; 95% CI + 9.2 to + 22.2%; FDR-adjusted *p* = 0.004). High-gamma augmentation at the left anterior MFG reached the statistical significance and maximum at 720 ms and 790 ms after the 3rd phrase offset, respectively (Fig. 4c; maximum augmentation =  + 5.2%; 95% CI + 2.6 to + 7.7%; FDR-adjusted *p* = 0.007). The magnitude of left orbitofrontal high-gamma augmentation failed to reach the significance but reached the maximum at 380 ms after the 3rd phrase offset (Fig. 4d; maximum augmentation =  + 5.5%; 95% CI − 0.1 to + 12.7%; FDR-adjusted *p* = 0.13).

Compared to the left anterior, the left posterior prefrontal high-gamma activity rose earlier (Fig. 4). The rate of increase of high-gamma activity, as quantified by a slope, was maximized exactly at the 2nd phrase offset at the left posterior MFG (+ 25%/s; 95% CI + 14 to + 36%; Supplementary Fig. S1) and 220 ms after the 2nd phrase offset at the left posterior IFG (+ 22%/s; 95% CI + 11 to + 33%). Likewise, that was maximized at 410 ms after the 2nd phrase offset at the left anterior MFG (+ 14%/s; 95% CI + 10 to + 18%) and 390 ms after the 2nd phrase offset at the left orbitofrontal region (+ 21%/s; 95% CI + 13 to + 29%).

### Left prefrontal high-gamma modulations during comprehension of sentences beginning with a concrete phrase

Questions beginning with a concrete phrase rapidly *activated* the left posterior prefrontal regions right after the 1st phrase offset. Compared to the left anterior, the left posterior prefrontal high-gamma augmentation reached the maximum earlier. Specifically, the magnitude of high-gamma augmentation at the left posterior MFG reached the statistical significance at 100 ms after the 1st phrase offset and the maximum at 10 ms before the 2nd phrase offset (Fig. 4a; maximum augmentation =  + 10.6%; 95% CI + 6.2 to + 15.9%; FDR-adjusted *p* = 0.006). Likewise, that at the left posterior IFG reached the statistical significance at 550 ms after the 1st phrase offset and the maximum at 630 ms after the 2nd phrase offset (Fig. 3b; maximum augmentation =  + 13.0%; 95% CI + 6.2 to + 21.8%; FDR-adjusted *p* = 0.012). The magnitude of high-gamma augmentation reached the maximum at 710 ms after the 3rd phrase offset in the left anterior MFG (Fig. 4c; maximum augmentation =  + 3.7%; 95% CI − 0.3 to + 7.6%; FDR-adjusted *p* = 0.34) and at 840 ms after the 3rd phrase offset in the left orbitofrontal region (Fig. 4d; maximum augmentation =  + 9.0%; 95% CI + 1.9 to + 17.2%; FDR-adjusted *p* = 0.30).

Compared to the left anterior, the left posterior prefrontal high-gamma activity rose earlier (Fig. 4). The rate of increase of high-gamma activity was maximized at 220 ms after the 1st phrase offset in the left posterior MFG (+ 15%/s; 95% CI + 9 to + 22%; Supplementary Fig. S1), at 400 ms after the 1st phrase offset in the left posterior IFG (+ 16%/s; 95% CI + 9 to + 24%). Likewise, that was maximized at 460 ms after the 1st phrase offset in the left anterior MFG (+ 9%/s; 95% CI + 5 to + 12%) and at 290 ms before the 2nd phrase offset in the left orbitofrontal region (+ 13%/s; 95% CI + 2 to + 23%).

### Right orbitofrontal deactivation preceded left posterior prefrontal activation during sentence comprehension

In question trials beginning with a concrete phrase, the maximum high-gamma suppression at the right orbitofrontal region took place at 150 ms before the 1st phrase offset (Fig. 4e; maximum suppression =  − 8.7%; 95% CI − 12.1 to − 5.4%; FDR-adjusted *p* = 0.015). Such right orbitofrontal suppression preceded the onset of significant high-gamma augmentation at the left posterior MFG (Fig. 4a; 100 ms after the 1st phrase onset) and the left posterior IFG (Fig. 4b; 550 ms after the 1st phrase onset).

In question trials beginning with a *wh-*interrogative, the maximum high-gamma suppression at the right orbitofrontal region took place at 270 ms before the 2nd phrase offset (Fig. 4e; maximum suppression =  − 8.9%, 95% CI − 12.1 to − 5.7%; FDR-adjusted *p* = 0.020). Such right orbitofrontal suppression likewise preceded the onset of significant high-gamma augmentation at the left posterior MFG (Fig. 4a; 290 ms after the 2nd phrase offset) and left posterior IFG (Fig. 4b; 340 ms after the 2nd phrase offset).

### Neuronal modulations differed between sentence types

**Figure 5:**
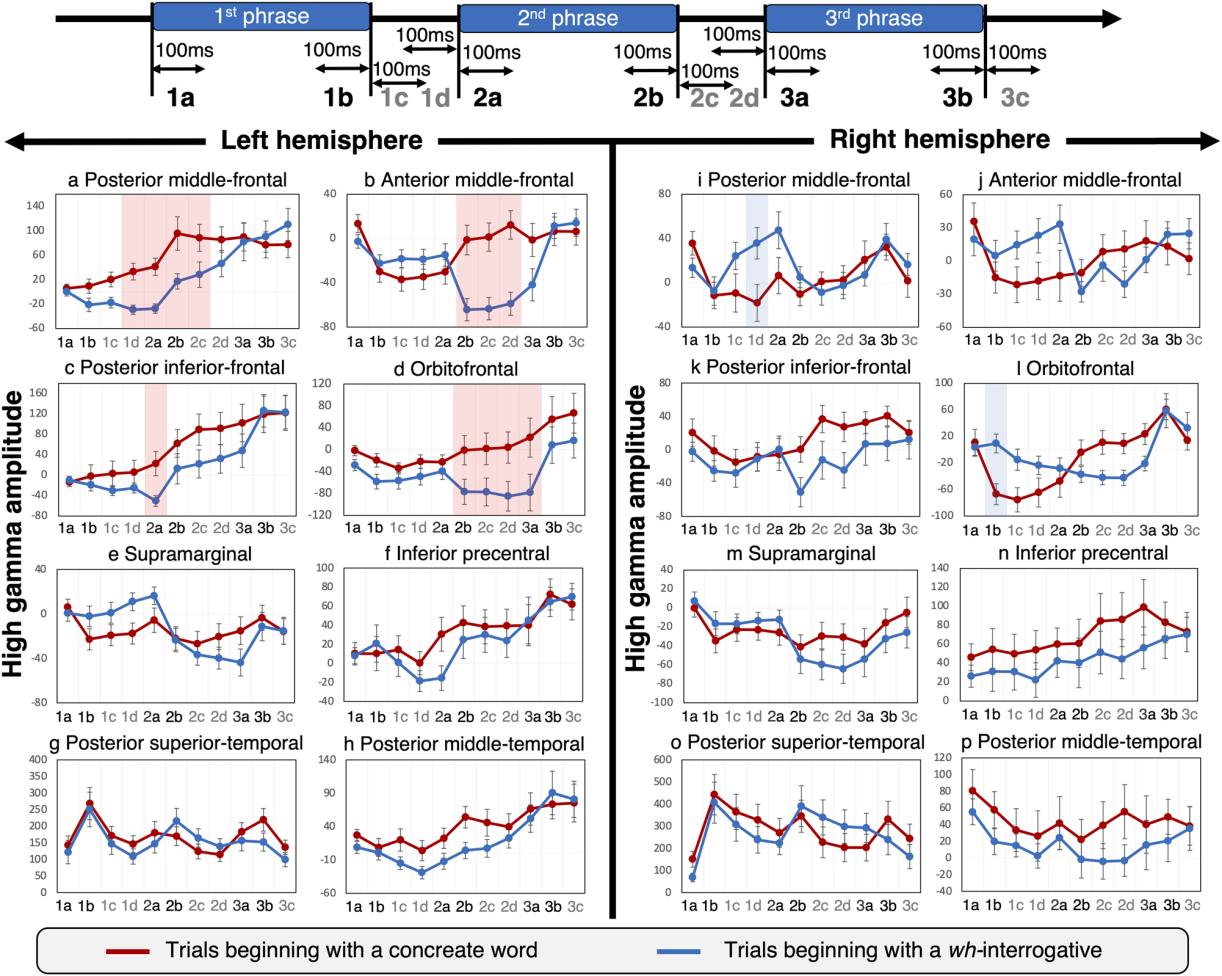
Phrase order-specific high-gamma modulations during sentence comprehension. (**a**) and (**c**) The left posterior middle- and inferior-frontal gyri (MFG and IFG) showed a rising of high-gamma activity around the 1st phrase offset during trials beginning with a concrete phrase. These regions showed high-gamma suppression maximally around the 1st phrase offset during trials beginning with a wh-interrogative. (**b**) and (**d**) The left anterior MFG and orbitofrontal regions showed high-gamma suppression maximally around the 2nd phrase offset during trials beginning with a wh-interrogative. (**i**) The right posterior MFG showed high-gamma suppression immediately before the 2nd phrase onset specifically during trials beginning with a concrete phrase. (**l**) The right orbitofrontal region showed high-gamma suppression immediately before the 1st phrase offset specifically during trials beginning with a concrete phrase.

The analysis of eleven 100-ms time windows (Fig. 5), each of which was time-locked to either phrase onset or offset, revealed that the spatiotemporal dynamics of high-gamma modulations differed between trials beginning with a *wh-*interrogative and those beginning with a concrete phrase. The left posterior prefrontal regions showed high-gamma modulations earlier than the left anterior prefrontal regions (Fig. 5). *After the 1st phrase offset,* compared to trials beginning with a *wh-*interrogative, those beginning with a concrete phrase showed higher high-gamma activity at the left posterior MFG (Fig. 5a; maximum difference across eleven 100-ms time windows was observed in 2b window: average difference in 2b =  + 78.4%; |t|= 3.48; 95% CI + 32.4 to + 124.5%; FDR-adjusted *p* = 0.02) and at the left posterior IFG (maximum difference [2a in Fig. 5c]: + 73.1%; |t|= 3.24; 95% CI + 26.9 to + 119.4%; FDR-adjusted *p* = 0.04). *Around the 2nd phrase offset,* likewise compared to trials beginning with a *wh-*interrogative, those beginning with a concrete phrase showed higher high-gamma activity at the left anterior MFG (maximum difference [2d in Fig. 5b]: + 71.2%; |t|= 5.59; 95% CI + 45.4 to + 97.1%; FDR-adjusted *p* = 0.0004) and at the left orbitofrontal (maximum difference [3a in Fig. 5d;]: + 100.2%; |t|= 3.92; 95% CI + 47.1 to + 153.4%; FDR-adjusted *p* = 0.01).

The earliest differential high-gamma modulation took place at the right orbitofrontal region *before the 1st phrase onset* (Fig. 5l); compared to trials beginning with a *wh-*interrogative, those starting with a concrete phrase showed smaller high-gamma activity (maximum difference [1b in Fig. 5l]: − 76.4%; |t|= 3.44; 95% CI − 123.3 to − 29.6%; FDR-adjusted *p* = 0.04). Likewise, compared to trials beginning with a *wh-*interrogative, those starting with a concrete phrase showed smaller high-gamma activity at the right posterior MFG (maximum difference [1d in Fig. 5i]: − 40.9%; |t|= 4.33; 95% CI − 60.5 to − 21.4%; FDR-adjusted *p* = 0.006) immediately after the 1st phrase onset.

### Mixed model analysis

The statistically significant high-gamma difference between sentence types during the 14 epochs based on the studentized bootstrap test (Fig. 5) remained significant even with the effects of patient and epilepsy profiles taken into account in the mixed model analysis (e.g., difference: − 68.7%; |t|= 4.71; 95% CI − 98.1 to − 39.1%; FDR-adjusted *p* = 0.0003 at the left posterior MFG during 100-ms period immediately after the 2nd phrase onset; Supplementary Table S3). We provided the statistical results, in detail, in Supplementary Tables S2–S15.

## Discussion

Our major observations include that neuronal deactivation, as reflected by suppression of high-gamma activity^8,24,27^, took place in the left posterior prefrontal regions around the 1st phrase offset only in trials beginning with a *wh*-interrogative (Fig. 4a,b). Such rapid left prefrontal deactivation cannot be attributed to the physical acoustic features of *wh*-interrogatives per se because *wh*-interrogatives were associated with left prefrontal activation in trials beginning with a concrete phrase (Fig. 3a,b).

Rapid left posterior prefrontal deactivation specific to trials beginning with a *wh*-interrogative is likewise difficult to attribute to the phenomenon of ’repetition suppression’, defined as a reduction in event-related neuronal activation when an identical stimulus is presented repeatedly. Prior iEEG studies commonly suggest that rapid event-related high-gamma activity elicited by repeated stimuli is smaller than that elicited by novel stimuli; thereby, the effect of such repetition suppression is most prominently seen in perceptual areas^33–35^. The present study indicated that the degree of high-gamma modulations in the STG of both hemispheres was similar between trials beginning with a *wh*-interrogative and a concrete phrase throughout the presentation of sentence stimuli (Fig. 5g,o). Our sentence stimuli included the following *wh*-interrogatives: ’nani-ga’ (*what* in English), ’doko-de’ (*where*), and ’itsu’ (*when*). *Wh*-interrogatives in Japanese do not contain a common phoneme, whereas those in English commonly include /w/. We designed the sentence comprehension task in which none of the consecutive questions included the same *wh*-interrogative. In trials beginning with a *wh-*interrogative, furthermore, rapid left posterior prefrontal high-gamma suppression around the 1st phrase offset (Fig. 4a,b) was followed by delayed left anterior prefrontal high-gamma suppression around the 2nd phrase offset (Fig. 4c,d). Thus, left prefrontal high-gamma suppression specific to *wh-*interrogatives reflects a cognitive process rather than a perceptual one. It may be difficult to attribute such early left prefrontal high-gamma suppression to the different processing loads or differences in binding/unification mechanisms across trials^36,37^ because such high-gamma suppression took place before the 2nd phrase onset.

We suggest that the *wh*-interrogative specific, rapid neuronal deactivation in the left posterior prefrontal regions would reflect a prioritization of the *bottom-up* processing of upcoming perceptual information in the STG by suppressing the *top-down* prediction or integration of lexical items. The present study showed that rapid left posterior prefrontal deactivation specific to a *wh*-interrogative took place simultaneously with sustained activation in the left posterior STG (Fig. 5g). A previous iEEG study reported a comparable observation that picture naming-related high-gamma suppression in the left IFG was temporally coupled with simultaneous high-gamma augmentation in the left occipital and posterior fusiform regions at 200–400 ms following the picture presentation^38^. A previous study reported that cathodal (i.e., inhibitory) transcranial direct current stimulation (tDCS) of the left dorsolateral prefrontal region transiently increased the attention to target visual stimuli, as rated by occipital event-related potentials^39^. Few studies suggest that the left posterior prefrontal regions consist of the default mode network, which is defined as the cortical areas showing neuronal activation during task disengagement but neuronal deactivation during the engagement^40^. The present study demonstrated that left posterior prefrontal high-gamma suppression was followed by prominent and sustained augmentation during the trials beginning with a *wh-*interrogative (Fig. 4a,b). Some consider that sentences beginning with *what* as the canonical word-order ones in Japanese^41^. If that is the case, early high-gamma suppression in the left posterior prefrontal region could be attributed to the facilitatory process for expected default word order.

Concrete phrase-specific, rapid high-gamma augmentation in the left posterior prefrontal regions may reflect the neuronal activation facilitating prediction and integration of the lexical items, in a *top-down* manner, based on their past experience of phrase usage^4^. Such a cognitive process is expected to help effortless determination of the semantic context expressed by a spoken sentence^16,42^. In other words, hearing a concrete phrase may subconsciously facilitate the internal selection of semantically-compatible lexical candidates for effortless verbal conversation^43^. In general, a particular verb is coupled with an adverb or object in many languages; for example, an adverb ’in the sky’ is directly combined with ’fly’ by far more frequently than ’eat’ in English (https://scholar.google.com/). Studies of patients with epilepsy and healthy individuals commonly reported that a verb generation task elicits hemodynamic activation in the left posterior prefrontal regions, including the IFG and MFG^44–46^. Lesion-deficit studies reported that damages involving the left posterior prefrontal regions impair verb generation^47,48^. Studies of healthy individuals reported that tDCS-based functional facilitation of the areas proximal to the left posterior prefrontal region transiently improved the learning of verbal and nonverbal association probability^49,50^.

The aforementioned interpretation for concrete phrase-specific, rapid left posterior prefrontal activation is consistent with previous iEEG literature. iEEG studies reported that auditory presentation of a single concrete word elicited high-gamma augmentation in the left posterior prefrontal regions maximally around the word offset^51,52^. iEEG studies also reported that visual presentation of a concrete word produced high-gamma augmentation in the left posterior prefrontal areas, including the IFG and MFG, at a post-stimulus latency of 300 ms^53,54^. Thereby, the spatial extent of such high-gamma augmentation was overlapped mostly with hemodynamic activation on fMRI^53^.

Our observations of the temporal order of high-gamma modulations support the general notion that the right anterior prefrontal cortex consistently exerts an inhibitory control and that the obliteration of the inhibitory function effectively facilitates the lexical prediction process by the left prefrontal region^18–22^. We specifically found that, in trials beginning with a concrete phrase, high-gamma suppression took place in the right orbitofrontal region around the 1st phrase offset (Fig. 4e). Such right orbitofrontal suppression preceded high-gamma augmentation in the left posterior MFG and IFG (Fig. 4a,b). In trials beginning with a *wh*-interrogative, high-gamma suppression took place in the right orbitofrontal region around the 2nd phrase offset (Fig. 4e). Such right orbitofrontal deactivation likewise preceded high-gamma augmentation in the left posterior MFG and IFG (Fig. 4a,b). In other words, regardless of the phrase order, the first heard concrete phrase deactivated the right orbitofrontal region and then activated the left posterior MFG and IFG. Studies using transcranial magnetic stimulation demonstrated that inhibition of local function facilitated the function of the contralateral homolog area presumably via transcallosal mediations^55,56^.

We found that high-gamma augmentation and suppression in the left posterior MFG and IFG preceded those in the left anterior MFG and orbitofrontal region (Fig. 5). High-gamma activity in the left anterior MFG and orbitofrontal region was augmented when phrase unification needed to occur between two concrete phrases and not between a *wh-*interrogative and concrete phrase. Thus, we suggest that the lexical processing of a single concrete phrase would take place initially in the left posterior prefrontal regions and that the integral processing of multiple concrete words would require the function of the more anterior prefrontal regions. We also suggest that left anterior MFG and orbitofrontal high-gamma augmentation may reflect semantic more than syntactic unification because a combination of a *wh-*interrogative and a concrete phrase did not elicit high-gamma augmentation. Our interpretation is consistent with the results of fMRI-based analysis indicating the presence of a functional gradient within the left prefrontal region in a posterior-dorsal to anterior-ventral direction^23,57^. Tasks requiring syntactic unification increased hemodynamic activation in the left posterior prefrontal regions, whereas those requiring semantic unification did in the more anterior and ventral regions, including the orbitofrontal gyrus^23^. A recent fMRI study suggests that distributional knowledge in the left orbitofrontal gyrus may exert semantic unification^43^.

We employed a mixed model analysis to increase the generalizability of our iEEG-based brain mapping. The results indicated that a within-individual difference in high-gamma dynamics between different sentence types remained significant even with the effects of patient and epilepsy profiles taken into account in the mixed model analysis (the detailed statistical results are provided in Supplementary Tables S2–S15). Sensory-related high-gamma dynamics have been suggested to be similar between non-epileptic areas of patients with focal epilepsy and the healthy cortex of nonhuman primates^25,58^. The present study excluded any electrode sites affected by the seizure onset zone, interictal spiking, or structural lesions from the analysis. Thus, our observed difference in high-gamma dynamics between different sentence types is difficult to attribute to the variance of epilepsy-related factors.

The inevitable limitations of our study include limited spatial sampling. The left supramarginal region of interest (ROI) had 57 electrode sites; with a beta of 0.8 and alpha of 0.05, our analysis can detect an effect size of 0.77. Conversely, the right posterior MTG had only 15. This observation indicates that the statistical power of the former ROI for detecting high-gamma modulations was approximately 1.8-times greater than that of the latter. Since the spatial extent of intracranial electrode placement was determined strictly by the clinical needs, most of the patients had iEEG sampling only from one hemisphere in the present study. Further studies using stereotactic EEG recordings are warranted to measure the spatiotemporal dynamics of the neural activity of the deep cortex, such as the insula. The causal relationship between two regions of interest may be further clarified by network analyses in the future^59,60^.

The present study included monolingual patients speaking Japanese, which is characterized by free word order^16^. It remains uncertain if the results of the present study are generalizable to individuals who speak other languages. Our previous study of English-speaking patients was not designed to determine the dynamics of high-gamma modulations during the offset of each phrase^38^. We plan to determine whether questions beginning with a *wh*-interrogative *specifically* deactivate the left posterior prefrontal regions in patients speaking English and other languages.

## Methods

### Participants

The inclusion criteria consisted of native Japanese-speaking patients with drug-resistant focal epilepsy who underwent an auditory sentence comprehension task (Fig. 2) during extraoperative iEEG recording at the National Center of Neurology and Psychiatry, Tokyo, Japan, and Tohoku University Hospital, Sendai, Japan. The exclusion criteria consisted of (i) inability to complete the task, (ii) brain malformations deforming the central or lateral sulcus, (iii) history of previous resective epilepsy surgery, and (iv) right-hemispheric language dominance as suggested by either the result of the Wada test or left-handedness associated with left-hemispheric congenital neocortical lesions^13–15,30^. The intelligence quotient (IQ) was measured with the Wechsler Intelligence Scale before the surgery (Table 1). This study was approved by ethical committees of Tohoku University Graduate School of Medicine and the National Center of Neurology and Psychiatry. We performed the analysis under the published guidelines^61^. Informed consent was obtained from a given patient or the guardian of a pediatric patient.

### Intracranial electrode placement and extraoperative iEEG recording

We acquired and analyzed iEEG data using methods similar to those reported previously^8,61^. Based on the results of non-invasive epilepsy evaluations, including seizure semiology, scalp video-EEG, and MRI, we implanted subdural platinum electrodes on the affected hemisphere (center-to-center distance: 5–10 mm). For localization of the epileptogenic zone to be surgically resected, we continuously recorded iEEG signals directly from the surface of the cerebral cortex with a sampling rate of 1,000 Hz (Nihon Kohden EEG 1200, Nihon Kohden, Tokyo, Japan). Excluded from further analyses were electrode sites classified as seizure onset zones responsible for habitual seizures, irritative zones generating interictal spike discharges, and those affected by structural lesions or artifacts^61,62^.

We created a 3D MR image with the location of subdural electrodes displayed directly on the brain surface^8,63^. We confirmed the spatial accuracy of electrode display by visual assessment of intraoperative pictures^64^. Using the FreeSurfer scripts (http://surfer.nmr.mgh.harvard.edu), we spatially normalized the locations of individual electrode sites to the Talairach coordinate and displayed all electrode sites on the FreeSurfer’s average surface image^8,65^. We employed an automatic parcellation of cortical gyri at both individual and spatially normalized brain surfaces^8,66^ and assigned an anatomical ROI to each electrode site. In the present study, we defined the posterior prefrontal region as the summation of the posterior MFG (Fig. 4a) and posterior IFG (Fig. 4b). We defined the anterior prefrontal region as the anterior MFG (Fig. 4c) and orbitofrontal region (including the pars orbitalis of the IFG and lateral orbitofrontal gyrus; Fig. 4d). We provided the number of available electrodes within each ROI in Table 2.

### Auditory sentence comprehension task

Patients underwent the task during extraoperative iEEG recording at the bedside during wakefulness. None of the patients had a seizure within two hours before the task. Each patient was instructed to overtly answer each of the 96 auditory sentence questions verbalized by a native-speaking male and presented via a speaker (Fig. 2). Each sentence was characterized by one of the two distinct phrase orders. The duration of each sentence was 1.8 s in total.

(i) Trials beginning with a concrete phrase: In 48 trials, a sentence question began with a concrete phrase; namely, it consisted of [adverb or object] followed by [verb] and [*wh-*interrogative]. In the present study, concrete phrases included [adverb or object] and [verb]. [adverb or object] began with a concrete noun because an adposition follows a noun in Japanese but precedes in English (Supplementary Tables S16 and S17). [*wh-*interrogative] included in a sentence was either ’*what*’ (16 trials), ’*when*’ (16 trials), or ’*where*’ (16 trials). The duration of the 1st phrase was 546 ± 80 ms (average ± SD), that between the 1st and 2nd phrases was 168 ± 57 ms, that of the 2nd phrase was 538 ± 83 ms, that between the 2nd and 3rd phrases was 155 ± 49 ms, and that of the 3rd phrase was 410 ± 50 ms.

(ii) Trials beginning with a *wh-*interrogative: In the other 48 trials, a sentence question began with [*wh-*interrogative] followed by [adverb or object] and [verb]. Using the National Institute for Japanese Language and Linguistics Database (https://pj.ninjal.ac.jp/corpus_center/bccwj/en/), *we controlled the number of syllables as well as the frequency of occurrence* between sentences beginning with [adverb or object] and [*wh-*interrogative] (*p* > 0.05 on one-way ANOVA between subjects). The duration of the 1st phrase was 436 ± 56 ms, that between the 1st and 2nd phrases was 181 ± 62 ms, that of the 2nd phrase was 487 ± 100 ms, that between the 2nd and 3rd phrases was 157 ± 60 ms, and that of the 3rd phrase was 554 ± 75 ms. We furthermore counterbalanced the sentence type across patients; this procedure was possible because a question with the same meaning can be expressed flexibly in a different order, as mentioned above (Fig. 2). We presented two different types of sentences in a pseudorandom order, whereas none of the consecutive trials contained the same [*wh-*interrogative]. None of the patients were informed that they would be asked questions with different types of word orders. We defined the response time as the period between the sentence offset (i.e., 3rd phrase offset) and response onset (Fig. 2). We excluded trials from further analysis if a patient failed to verbalize a correct answer overtly. We determined if the median response time of given patients differed between trials beginning with a concrete phrase and those starting with a *wh*-interrogative (Wilcoxon Signed Rank Test).

### Measurement of event-related high-gamma modulations

We measured event-related high-gamma activity using a method similar to those previously reported^8,67^. We transformed iEEG signals into the time–frequency domain, in steps of 10 ms and 5 Hz, using a complex demodulation technique^68^ incorporated in the BESA EEG software package (BESA GmbH, Gräfelfing, Germany^69^). At each 10-ms/5-Hz time–frequency bin at each electrode site, we measured the percent change of high-gamma amplitude (65–95 Hz) relative to the mean during a resting/baseline period between − 600 and − 200 ms relative to sentence onset (Fig. 2). *The frequency of alternating current (AC) is 50 Hz in Eastern Japan and 60 Hz in Western Japan*; thus, the aforementioned high-gamma band signals were least affected by AC-related artifacts^67^. We created an animation movie (Supplementary Videos S1 and S2)^8^ presenting the changes in high-gamma amplitude at all electrode sites on the average FreeSurfer pial surface image with a Gaussian half-width at half-maximum of 7.5 mm.

### Statistical analysis

We plotted the mean and standard error (SE) of high-gamma amplitude change across all available electrode sites at each of the 16 regions of interest (ROIs) as a function of time (Fig. 4). In the resulting plots, we indicated the epochs showing statistically-significant augmentation (or suppression) of high-gamma activity at given ROIs during two types of trials, based on the permutation test followed by an FDR correction for multiple comparisons across time windows (Fig. 4). Specifically, a permutation test (n = 1000) evaluated the null hypothesis that the population mean of high-gamma modulations at a given time point would be equal to zero with a two-sided 5% significance level, followed by FDR correction across the time window (i.e., 451 bins for 4,500 ms). After the FDR correction, in Fig. 4, we provided horizontal red and blue bars to visualize the periods showing a significant high-gamma modulation lasting at least 60 ms. We likewise computed the slope of high-gamma amplitude modulation at a given 600-ms time sliding window, to determine when the rate of rising of high-gamma activity was maximized at a given ROI during a given trial type (Supplementary Fig. S1).

A studentized bootstrap test determined at what time windows and at what ROIs high-gamma amplitudes differed between trials beginning with a concrete phrase and a *wh-*interrogative. We repeated the tests at all eleven 100-ms time windows (Fig. 5) set around phrase onsets and offsets and performed an FDR correction for multiple time windows and ROIs.

We subsequently employed a mixed model analysis to determine whether the difference in high-gamma amplitude between sentence types would remain significant even with the effects of the following predictor variables taken into account. The dependent variable was the high-gamma amplitude at a given 100-ms time window at a given ROI. The fixed effect predictors included (i) phrase order type, (ii) patient age, (iii) age of epilepsy onset, (iv) sex, (v) number of oral antiepileptic drugs (reflecting the severity of seizure burden^70^), (vi) full-scale intelligence quotient (FIQ), and (vii) the presence of a congenital lesion (e.g., focal cortical dysplasia). The random factors included the intercept and the patient. We performed an FDR correction for multiple comparisons (Supplementary Tables S2–S15).

### Ethics approval

This study was approved by ethical committees of Tohoku University Graduate School of Medicine and the National Center of Neurology and Psychiatry. We performed the analysis under the published guidelines ^61^. Informed consent was obtained from a given patient or the guardian of a pediatric patient.

## Supplementary Information

Supplementary Video 1.

Supplementary Video 2.

Supplementary Information 1.

## Data Availability

The data from the present study, including clinical information, iEEG, and MRI, as well as MATLAB-based in-house software are available upon request to the corresponding author (E.A.).

## Abbreviations

AC: Alternating current
BA: Brodmann area
FIQ: Full-scale intelligence quotient
fMRI: Functional MRI
iEEG: Intracranial EEG
IFG: Inferior-frontal gyrus
MFG: Middle-frontal gyrus
MTG: Middle-temporal gyrus
ROI: Region of interest
STG: Superior-temporal gyrus
tDCS: Transcranial direct current stimulation
