# Supplementary material for: Your verbal questions beginning with 'what' will rapidly deactivate the left prefrontal cortex of listeners
Source: Sci Rep. 2021 Mar 4;11:5257. doi: 10.1038/s41598-021-84610-1 (PMC7933162; doi:10.1038/s41598-021-84610-1)
Supplement: Supplementary file 3 — Supplementary Information 1. [file 41598_2021_84610_MOESM3_ESM.docx]

**Supplementary document in**

**Your verbal questions beginning with '*what*' will rapidly deactivate the left prefrontal cortex of listeners**

**Hirotaka Iwaki^1,3,+^, Masaki Sonoda^1,6,+^, Shin-ichiro Osawa^3,4,*^, Brian H. Silverstein^9^,**

**Takumi Mitsuhashi^1,10^, Kazushi Ukishiro^3,6^, Yutaro Takayama^3,6,7^, Toshimune Kambara^1,8^,**

**Kazuo Kakinuma^5^, Kyoko Suzuki^5^, Teiji Tominaga^4^, Nobukazu Nakasato^3^, Masaki Iwasaki^7,*^,**

**and Eishi Asano^1,2,*^**

^1^Department of Pediatrics, Children's Hospital of Michigan, Wayne State University, Detroit, Michigan, 48201, USA.

^2^Department of Neurology, Children's Hospital of Michigan, Wayne State University, Detroit, Michigan, 48201, USA.

^3^Department of Epileptology, Tohoku University Graduate School of Medicine, Sendai, 9808575, Japan.

^4^Department of Neurosurgery, Tohoku University Graduate School of Medicine, Sendai, 9808575, Japan.

^5^Department of Behavioral Neurology and Cognitive Neuroscience, Tohoku University Graduate School of Medicine, Sendai, 9808575, Japan.

^6^Department of Neurosurgery, Graduate School of Medicine, Yokohama City University, Kanagawa, 2360004, Japan.

^7^Department of Neurosurgery, National Center Hospital, National Center of Neurology and Psychiatry, Tokyo, 1878551, Japan.

^8^Department of Psychology, Hiroshima University, Hiroshima, 7398524, Japan.

^9^Translational Neuroscience Program, Wayne State University, Detroit, Michigan, 48201, USA.

^10^Department of Neurosurgery, Juntendo University, School of Medicine, Tokyo, 1138421, Japan.

^+^these authors contributed equally to this work.

^*^corresponding. Shin-ichiro Osawa: [osawa@nsg.med.tohoku.ac.jp](mailto:osawa@nsg.med.tohoku.ac.jp), Masaki Iwasaki: [iwa@ncnp.go.jp](mailto:iwa@ncnp.go.jp), and Eishi Asano: [easano@med.wayne.edu](mailto:easano@med.wayne.edu)

**This supplementary document includes the legend for Videos S1-S2, Figure S1, and Tables S1-S17.**

**Video Legends**

**Supplementary Video S1. Spatiotemporal dynamics of high-gamma modulations during sentence comprehension task.** The animation video demonstrates the spatiotemporal dynamics of high-gamma modulations during sentence comprehension task. 0 ms: Onset of auditory sentence question. Left: Trials assigning questions consisting of [adverb or object] followed by [verb] and [*wh-*interrogative]. Right: Trials assigning questions consisting of [*wh-*interrogative] followed by [adverb or object] and [verb].

**Supplementary Video S2. Spatiotemporal dynamics of high-gamma modulations during sentence comprehension task (response onset).** The animation video demonstrates the spatiotemporal dynamics of high-gamma modulations during sentence comprehension task. 0 ms: Onset of overt response. Left: Trials assigning questions consisting of [adverb or object] followed by [verb] and [*wh-*interrogative]. Right: Trials assigning questions consisting of [*wh-*interrogative] followed by [adverb or object] and [verb].

**
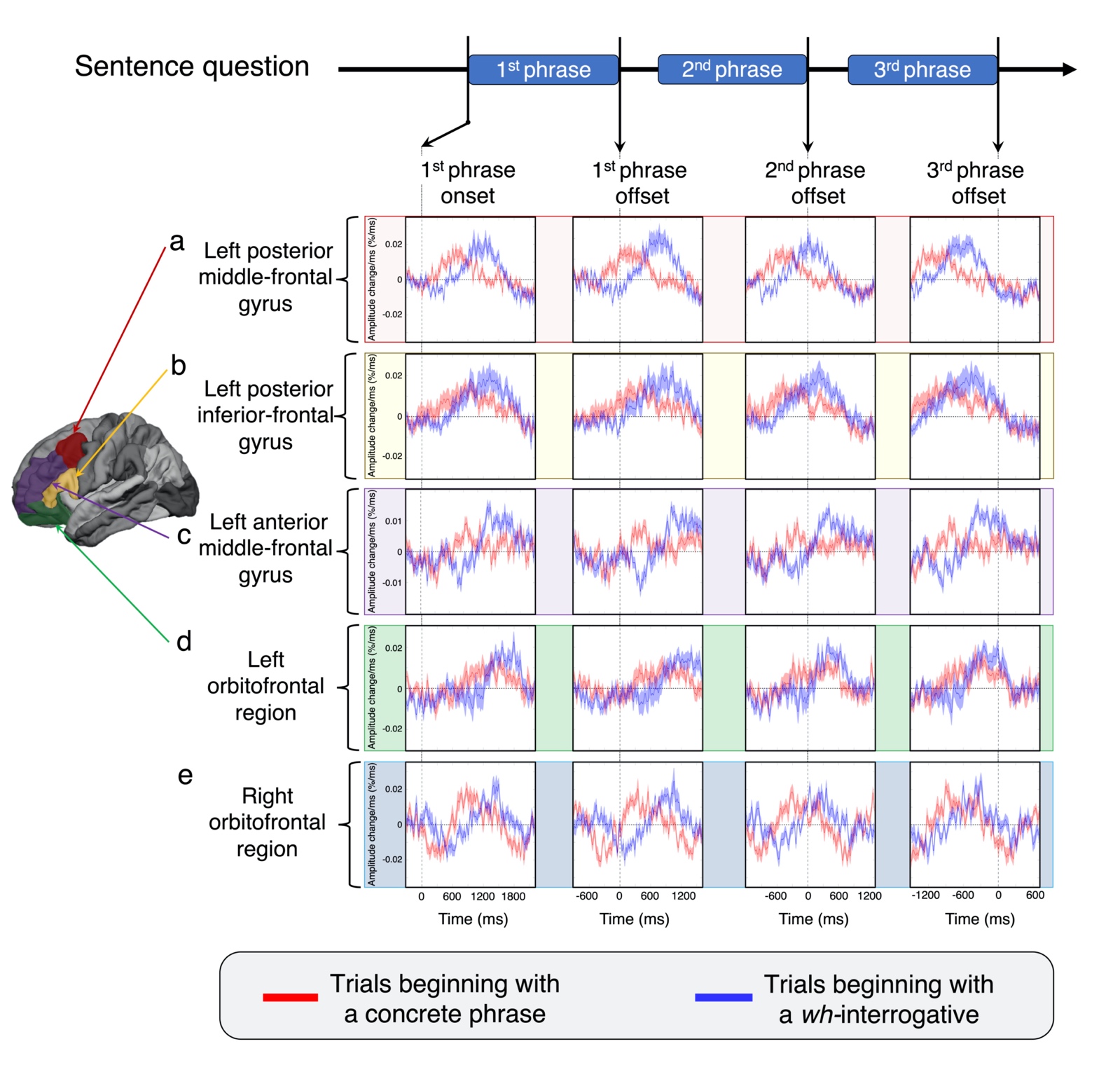
**

**Supplementary Figure S1. The slope of high-gamma modulation during sentence comprehension task.** We computed the slope of high-gamma amplitude modulation at a given 600-ms time sliding window, to determine when the rate of rising of high-gamma activity was maximized at a given regions of interest during a given trial type. (**a**)-(**d**) The slope of high-gamma modulation in the left hemisphere. (**e**) The slope of high-gamma modulation in the right orbitofrontal region. The mean slope presented with a standard error bar. Red plot: Trials beginning with a concrete phrase. Blue plot: Trials beginning with a *wh*-interrogative. First column: Time-locked to the 1st phrase onset (i.e., sentence onset). Second column: Time-locked to the 1st phrase offset. Third column: Time-locked to the 2nd phrase offset. Fourth column: Time-locked to the 3rd phrase offset (i.e., sentence offset). (**a**) and (**b**) The left posterior middle- and inferior-frontal gyri (MFG and IFG) showed the maximum slope around the 1st phrase offset during trials beginning with a concrete phrase. These regions showed the maximum slope around the 2nd phrase offset during trials beginning with a *wh*-interrogative. (**c**) and (**d**) The left anterior MFG and orbitofrontal regions showed the maximum slope immediately before the 2nd phrase offset during trials beginning with a concrete phrase. These regions showed the maximum slope immediately before the 3rd phrase offset during trials beginning with a *wh*-interrogative. (**e**) The right orbitofrontal region showed a negative slope before the 1st phrase offset during trials beginning with a concrete phrase and before the 2nd phrase offset during trials beginning with a *wh*-interrogative.

|  |  | Trials beginning with  a concrete phrase | | Trials beginning with  a *wh*-interrogative | |
| --- | --- | --- | --- | --- | --- |
| Patient number | Assigned questions | Median  response time (ms) | Proportion of correct answers | Median  response time (ms) | Proportion of correct answers |
| 1 | A | 1,976 | 100.0% | 1,795 | 99.0% |
| 2 | A | 2,305 | 100.0% | 2,837 | 100.0% |
| 3 | A | 1,043 | 99.0% | 1,204 | 99.0% |
| 4 | B | 1,941 | 95.8% | 1,695 | 100.0% |
| 5 | B | 1,199 | 89.6% | 980 | 85.4% |
| 6 | B | 2,718 | 96.9% | 3,305 | 99.0% |
| 7 | A | 1,488 | 100.0% | 2,612 | 99.0% |
| 8 | B | 3,661 | 93.8% | 3,599 | 97.9% |
| 9 | B | 1,325 | 99.0% | 1,254 | 99.0% |
| 10 | A | 2,115 | 100.0% | 2,545 | 99.0% |
| 11 | B | 5,473 | 93.8% | 4,310 | 91.7% |
| 12 | A | 4,638 | 99.0% | 4,007 | 97.9% |
| 13 | B | 2,389 | 97.9% | 2,104 | 97.9% |
| 14 | A | 1,110 | 100.0% | 960 | 100.0% |
| 15 | A | 6,660 | 88.5% | 6,371 | 89.6% |
| 16 | B | 901 | 97.9% | 883 | 100.0% |
| 17 | A | 2,009 | 95.8% | 2,091 | 99.0% |
| 18 | B | 773 | 100.0% | 576 | 100.0% |
| 19 | A | 1,077 | 100.0% | 1,230 | 100.0% |
| 20 | A | 1,347 | 99.0% | 1,215 | 97.9% |
| 21 | B | 760 | 100.0% | 756 | 100.0% |
| 22 | B | 1,359 | 91.7% | 1,095 | 89.6% |
| 23 | B | 700 | 100.0% | 641 | 100.0% |

**Supplementary Table S1.** Median response time (ms) and the proportion of included trials in a given patient. **Table S16** shows the list of Type-A questions used in the present study. **Table S17** shows the list of Type-B questions.
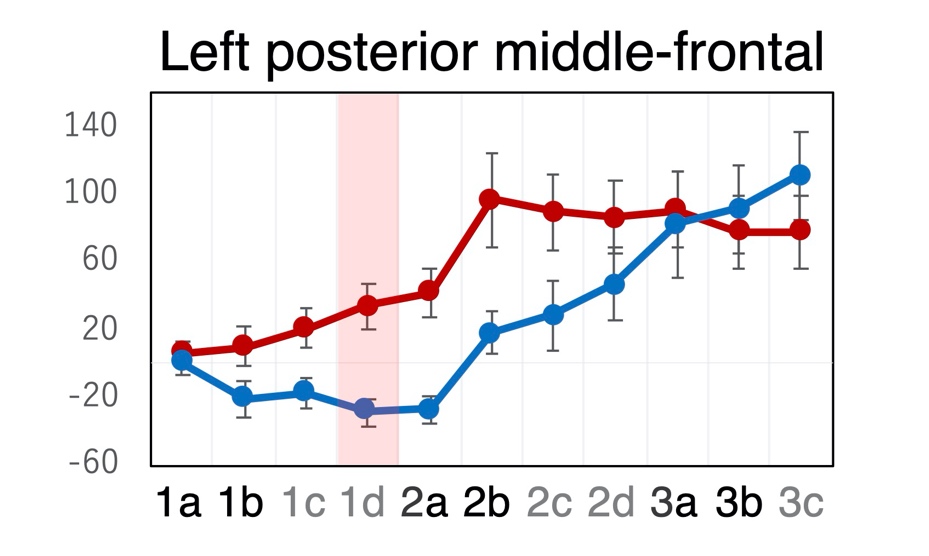


| Parameters | Estimate | SE | \|t\| | Unadjusted *p* | 95%CI LL | 95%CI UL |
| --- | --- | --- | --- | --- | --- | --- |
| Age | -4.5 | 3.4 | 1.3 | 0.20 | -11.3 | 2.4 |
| Age of epilepsy onset | 4.7 | 89.0 | 0.5 | 0.60 | -13.2 | 22.7 |
| Male | 48.5 | 24.4 | 2.0 | 0.05 | -0.4 | 97.3 |
| Number of antiepileptic drugs, n | 0* | . | . | . | . | . |
| FIQ | 0* | . | . | . | . | . |
| Absence of cortical lesion on MRI | 0* | . | . | . | . | . |
| Trials beginning with a *wh*-interrogative | -62.4 | 14.7 | 4.2 | 0.00008 | -91.8 | -32.9 |

**Supplementary Table S2.** Results of mixed model analysis in the left posterior middle-frontal gyrus during the 100-ms period immediately before the 2^nd^ phrase onset (period highlighted in the plot above). SE = Standard error, CI = Confidence interval, FIQ = Full-scale intelligence quotient, LL = Lower limit, UL = Upper limit.

* This parameter is set to zero because it is redundant. Compared to trials beginning with a *wh*-interrogative, those beginning with a concrete phrase was associated with 62% greater high gamma activity during the 100-ms period immediately before the 2^nd^ phrase onset.


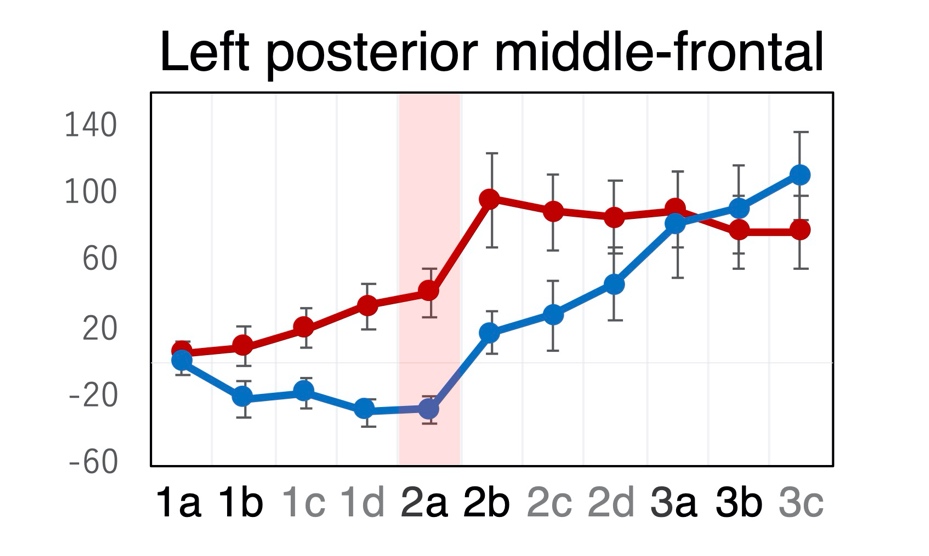


| Parameters | Estimate | SE | \|t\| | Unadjusted *p* | 95%CI LL | 95%CI UL |
| --- | --- | --- | --- | --- | --- | --- |
| Age | -6.4 | 3.4 | 1.9 | 0.07 | -13.2 | 0.5 |
| Age of epilepsy onset | 8.5 | 9.0 | 0.9 | 0.35 | -9.5 | 26.5 |
| Male | 51.0 | 24.5 | 2.1 | 0.07 | -2.0 | 100.0 |
| Number of antiepileptic drugs, n | 0* | . | . | . | . | . |
| FIQ | 0* | . | . | . | . | . |
| Absence of cortical lesion on MRI | 0* | . | . | . | . | . |
| Trials beginning with a *wh*-interrogative | -68.7 | 14.7 | 4.7 | 0.00002 | -98.1 | -39.1 |

**Supplementary Table S3.** Results of mixed model analysis in the left posterior middle-frontal gyrus during the 100-ms period immediately after the 2nd phrase onset (period highlighted in the plot above). SE = Standard error, FIQ = Full-scale intelligence quotient, CI = Confidence interval, LL = Lower limit, UL = Upper limit.

* This parameter is set to zero because it is redundant. Compared to trials beginning with a *wh*-interrogative, those beginning with a concrete phrase was associated with 69% greater high gamma activity during the 100-ms period immediately after the 2^nd^ phrase onset.


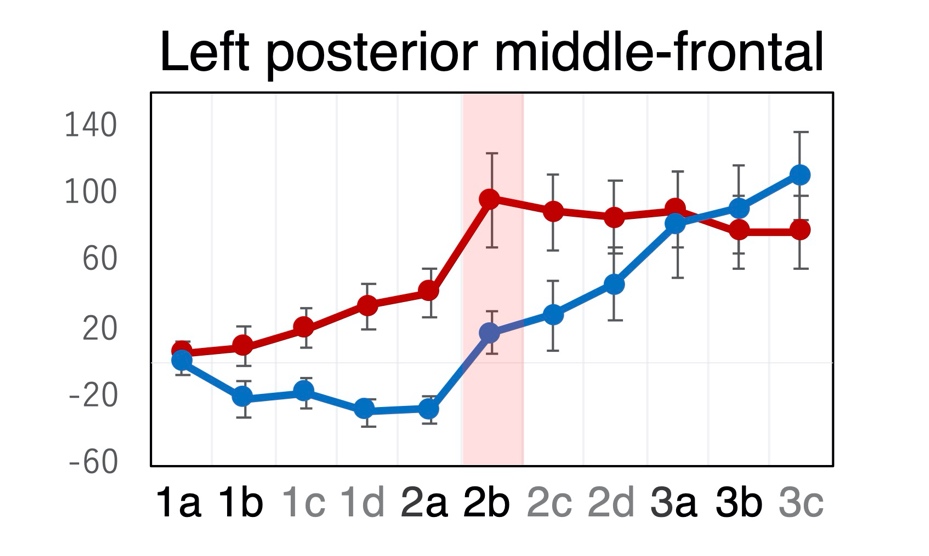


| Parameters | Estimate | SE | \|t\| | Unadjusted *p* | 95%CI LL | 95%CI UL |
| --- | --- | --- | --- | --- | --- | --- |
| Age | -17.4 | 6.2 | 2.8 | 0.01 | -29.7 | -5.0 |
| Age of epilepsy onset | 11.1 | 16.2 | 0.7 | 0.49 | -21.3 | 43.5 |
| Male | 121.9 | 44.0 | 2.8 | 0.32 | -33.8 | 201.0 |
| Number of antiepileptic drugs, n | 0* | . | . | . | . | . |
| FIQ | 0* | . | . | . | . | . |
| Absence of cortical lesion on MRI | 0* | . | . | . | . | . |
| Trials beginning with a *wh*-interrogative | -78.4 | 26.5 | 3.0 | 0.004 | -131.5 | -25.4 |

**Supplementary Table S4.** Results of mixed model analysis in the left posterior middle-frontal gyrus during the 100-ms period immediately before the 2^nd^ phrase offset (period highlighted in the plot above). SE = Standard error, FIQ = Full-scale intelligence quotient, CI = Confidence interval, LL = Lower limit, UL = Upper limit.

* This parameter is set to zero because it is redundant. Compared to trials beginning with a *wh*-interrogative, those beginning with a concrete phrase was associated with 78% greater high gamma activity during the 100-ms period immediately before the 2^nd^ phrase offset.


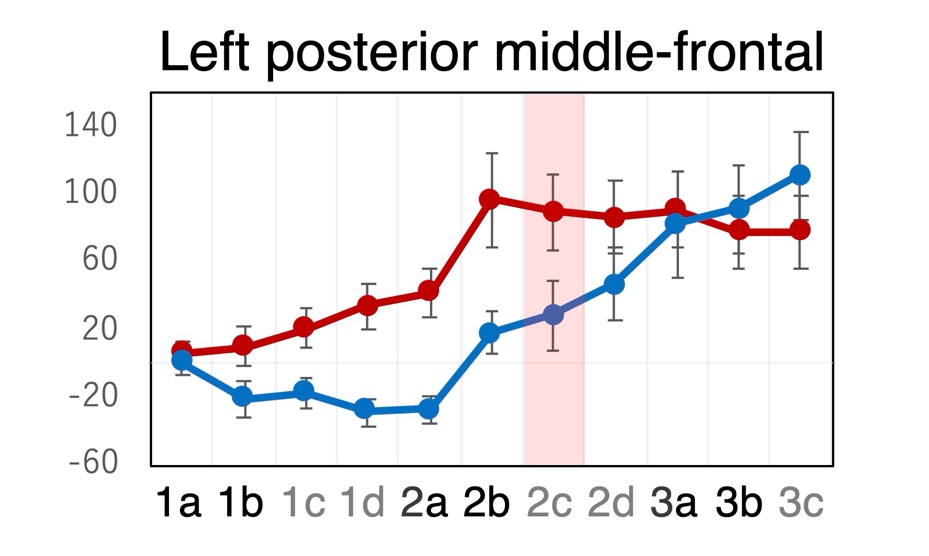


| Parameters | Estimate | SE | \|t\| | Unadjusted *p* | 95%CI LL | 95%CI UL |
| --- | --- | --- | --- | --- | --- | --- |
| Age | -20.7 | 5.7 | 2.8 | 0.001 | -32.1 | -9.3 |
| Age of epilepsy onset | 7.7 | 15.0 | 0.7 | 0.61 | -22.2 | 37.6 |
| Male | 161.3 | 40.7 | 2.8 | 0.21 | -79.9 | 242.8 |
| Number of antiepileptic drugs, n | 0* | . | . | . | . | . |
| FIQ | 0* | . | . | . | . | . |
| Absence of cortical lesion on MRI | 0* | . | . | . | . | . |
| Trials beginning with a *wh*-interrogative | -60.4 | 24.5 | 3.0 | 0.02 | -109.5 | -11.3 |

**Supplementary Table S5.** Results of mixed model analysis in the left posterior middle-frontal gyrus during the 100-ms period immediately after the 2^nd^ phrase offset (period highlighted in the plot above). SE = Standard error, FIQ = Full-scale intelligence quotient, CI = Confidence interval, LL = Lower limit, UL = Upper limit.

* This parameter is set to zero because it is redundant. Compared to trials beginning with a *wh*-interrogative, those beginning with a concrete phrase was associated with 60% greater high gamma activity during the 100-ms period immediately after the 2^nd^ phrase offset.


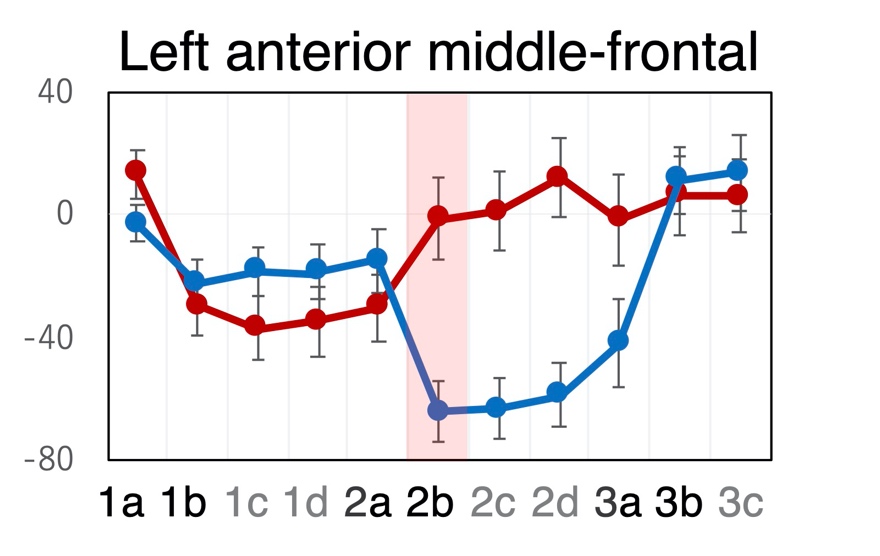


| Parameters | Estimate | SE | \|t\| | Unadjusted *p* | 95%CI LL | 95%CI UL |
| --- | --- | --- | --- | --- | --- | --- |
| Age | 8.7 | 8.7 | 1.0 | 0.32 | -8.7 | 26.1 |
| Age of epilepsy onset | 3.0 | 5.8 | 0.5 | 0.61 | -8.7 | 14.6 |
| Male | -199.0 | 216.2 | 0.9 | 0.36 | -630.6 | 232.5 |
| Number of antiepileptic drugs, n | -49.4 | 38.7 | 1.3 | 0.21 | -126.7 | 27.9 |
| FIQ | 0.8 | 1.9 | 0.4 | 0.68 | -3.0 | 4.6 |
| Absence of cortical lesion on MRI | 87.7 | 39.4 | 2.2 | 0.09 | -9.1 | 166.3 |
| Trials beginning with a *wh*-interrogative | -62.9 | 16.0 | 3.9 | 0.0002 | -94.9 | -30.9 |

**Supplementary Table S6.** Results of mixed model analysis in the left anterior middle-frontal gyrus during the 100-ms period immediately before the 2^nd^ phrase offset (period highlighted in the plot above). SE = Standard error, FIQ = Full-scale intelligence quotient, CI = Confidence interval, LL = Lower limit, UL = Upper limit.

Compared to trials beginning with a *wh*-interrogative, those beginning with a concrete phrase was associated with 63% greater high gamma activity during the 100-ms period immediately before the 2^nd^ phrase offset.


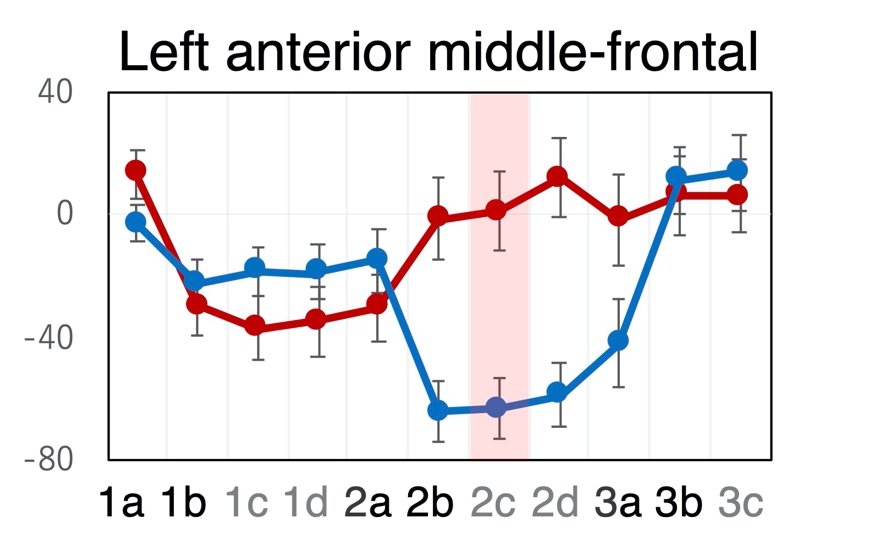


| Parameters | Estimate | SE | \|t\| | Unadjusted *p* | 95%CI LL | 95%CI UL |
| --- | --- | --- | --- | --- | --- | --- |
| Age | 15.3 | 8.5 | 1.8 | 0.07 | -1.5 | 32.2 |
| Age of epilepsy onset | 6.8 | 5.7 | 1.2 | 0.23 | -4.5 | 18.1 |
| Male | -351.1 | 209.1 | 1.7 | 0.10 | -768.4 | 66.1 |
| Number of antiepileptic drugs, n | -68.7 | 37.4 | 1.8 | 0.07 | -143.5 | 6.0 |
| FIQ | 2.0 | 1.9 | 1.1 | 0.27 | -1.7 | 5.7 |
| Absence of cortical lesion on MRI | 86.6 | 38.1 | 2.2 | 0.10 | -10.6 | 162.5 |
| Trials beginning with a *wh*-interrogative | -64.8 | 15.5 | 4.2 | 0.00009 | -95.7 | -33.8 |

**Supplementary Table S7.** Results of mixed model analysis in the left anterior middle-frontal gyrus during the 100-ms period immediately after the 2^nd^ phrase offset (period highlighted in the plot above). SE = Standard error, FIQ = Full-scale intelligence quotient, CI = Confidence interval, LL = Lower limit, UL = Upper limit.

Compared to trials beginning with a *wh*-interrogative, those beginning with a concrete phrase was associated with 65% greater high gamma activity during the 100-ms period immediately after the 2^nd^ phrase offset.


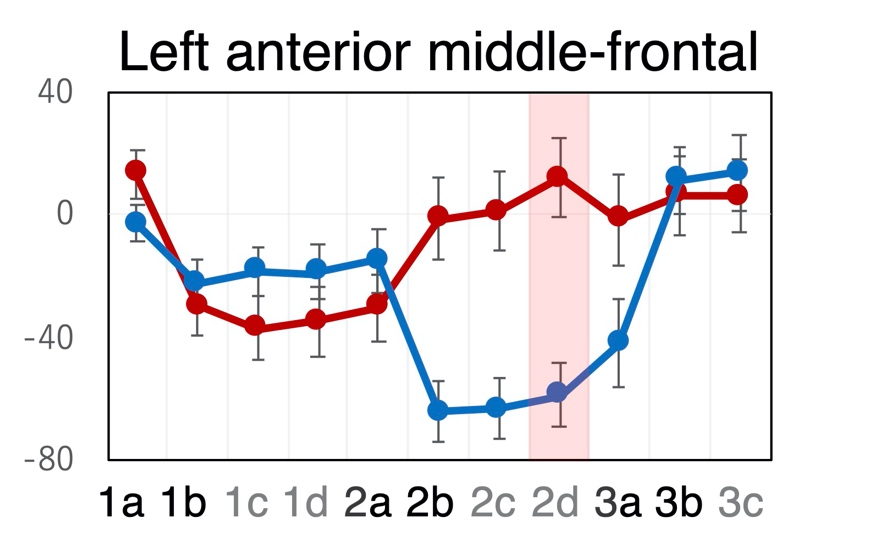


| Parameters | Estimate | SE | \|t\| | Unadjusted *p* | 95%CI LL | 95%CI UL |
| --- | --- | --- | --- | --- | --- | --- |
| Age | 14.6 | 8.4 | 1.7 | 0.09 | -2.1 | 31.3 |
| Age of epilepsy onset | 8.8 | 5.6 | 1.6 | 0.12 | -2.3 | 20.0 |
| Male | -356.1 | 207.2 | 1.7 | 0.09 | -769.7 | 57.4 |
| Number of antiepileptic drugs, n | -77.5 | 37.1 | 2.1 | 0.06 | -151.6 | 3.4 |
| FIQ | 2.2 | 1.8 | 1.2 | 0.23 | -1.5 | 5.9 |
| Absence of cortical lesion on MRI | 103.3 | 37.7 | 2.7 | 0.12 | -8.0 | 18.6 |
| Trials beginning with a *wh*-interrogative | -71.2 | 15.4 | 4.6 | 0.00002 | -101.9 | -40.6 |

**Supplementary Table S8.** Results of mixed model analysis in the left anterior middle-frontal gyrus during the 100-ms period immediately before the 3^rd^ phrase onset (period highlighted in the plot above). SE = Standard error, FIQ = Full-scale intelligence quotient, CI = Confidence interval, LL = Lower limit, UL = Upper limit.

Compared to trials beginning with a *wh*-interrogative, those beginning with a concrete phrase was associated with 71% greater high gamma activity during the 100-ms period immediately before the 3^rd^ phrase onset.


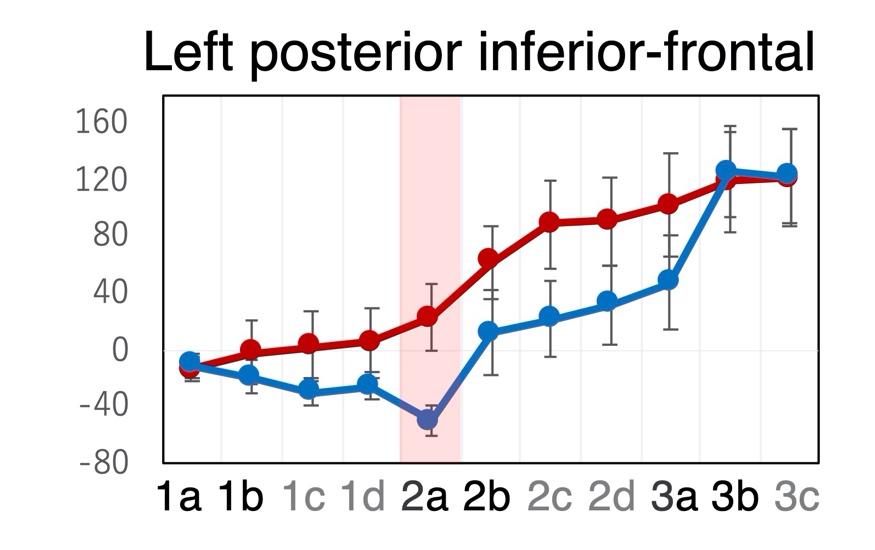


| Parameters | Estimate | SE | \|t\| | Unadjusted *p* | 95%CI LL | 95%CI UL |
| --- | --- | --- | --- | --- | --- | --- |
| Age | -4.0 | 6.6 | 0.6 | 0.55 | -17.3 | 9.2 |
| Age of epilepsy onset | -0.7 | 5.7 | 0.1 | 0.91 | -12.2 | 10.9 |
| Male | 87.9 | 159.0 | 0.6 | 0.58 | -231.1 | 406.8 |
| Number of antiepileptic drugs, n | -16.2 | 32.6 | 0.5 | 0.62 | -81.7 | 49.3 |
| FIQ | -1.8 | 1.5 | 1.2 | 0.24 | -4.9 | 1.3 |
| Absence of cortical lesion on MRI | 23.5 | 60.9 | 0.4 | 0.70 | -98.7 | 145.7 |
| Trials beginning with a *wh*-interrogative | -73.1 | 24.6 | 3.0 | 0.005 | -122.6 | -23.7 |

**Supplementary Table S9.** Results of mixed model analysis in the left posterior inferior-frontal gyrus during the 100-ms period immediately after the 2^nd^ phrase onset (period highlighted in the plot above). SE = Standard error, FIQ = Full-scale intelligence quotient, CI = Confidence interval, LL = Lower limit, UL = Upper limit.

Compared to trials beginning with a *wh*-interrogative, those beginning with a concrete phrase was associated with 73% greater high gamma activity during the 100-ms period immediately after the 2^nd^ phrase onset.


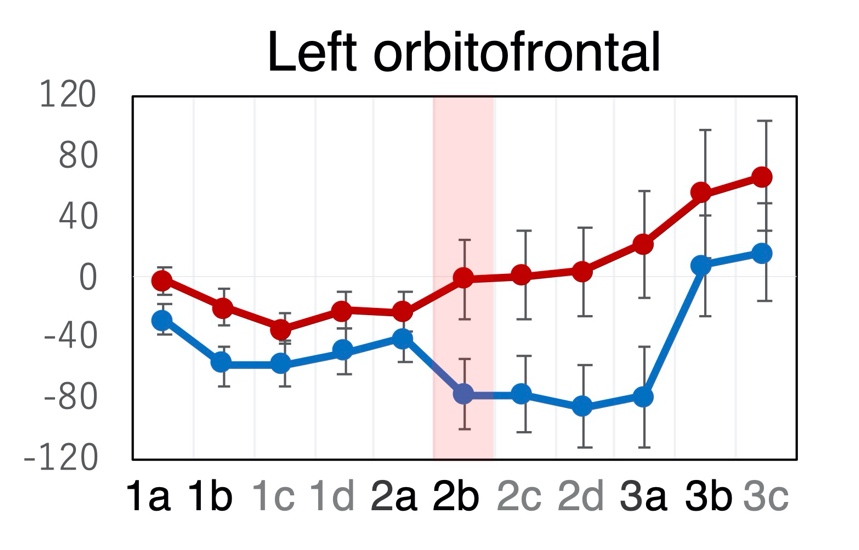


| Parameters | Estimate | SE | \|t\| | Unadjusted *p* | 95%CI LL | 95%CI UL |
| --- | --- | --- | --- | --- | --- | --- |
| Age | 3.9 | 19.1 | 0.2 | 0.84 | -34.8 | 42.6 |
| Age of epilepsy onset | -7.4 | 11.7 | 0.6 | 0.53 | -31.1 | 16.2 |
| Male | -54.0 | 443.7 | 0.1 | 0.90 | -953.0 | 845.0 |
| Number of antiepileptic drugs, n | -33.5 | 56.9 | 0.6 | 0.56 | -148.9 | 81.8 |
| FIQ | -9.2 | 3.7 | 2.5 | 0.18 | -16.7 | 1.7 |
| Absence of cortical lesion on MRI | 0* | . | . | . | . | . |
| Trials beginning with a *wh*-interrogative | -75.8 | 27.0 | 2.8 | 0.008 | -130.4 | -21.1 |

**Supplementary Table S10.** Results of mixed model analysis in the left orbitofrontal region during the 100-ms period immediately before the 2^nd^ phrase offset (period highlighted in the plot above). SE = Standard error, FIQ = Full-scale intelligence quotient, CI = Confidence interval, LL = Lower limit, UL = Upper limit.

* This parameter is set to zero because it is redundant. Compared to trials beginning with a *wh*-interrogative, those beginning with a concrete phrase was associated with 76% greater high gamma activity during the 100-ms period immediately before the 2^nd^ phrase offset.


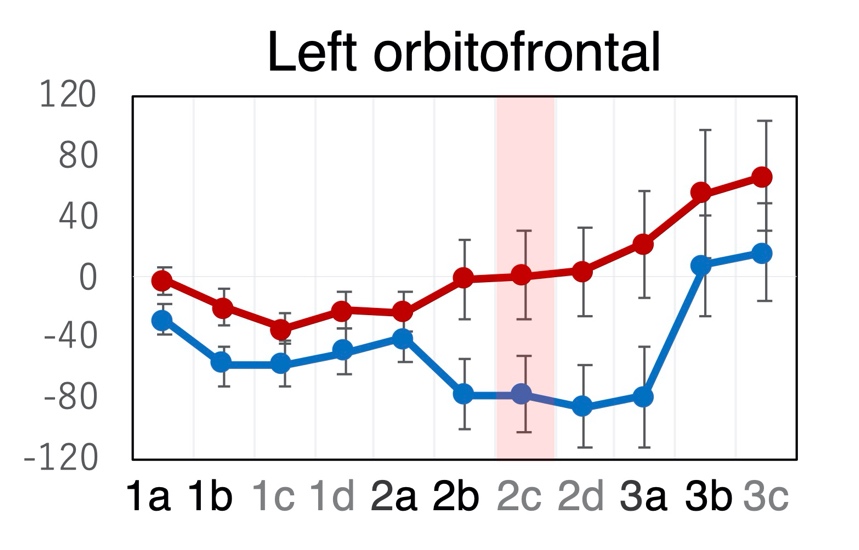


| Parameters | Estimate | SE | \|t\| | Unadjusted *p* | 95%CI LL | 95%CI UL |
| --- | --- | --- | --- | --- | --- | --- |
| Age | 0.5 | 21.2 | 0.0 | 0.98 | -42.4 | 43.5 |
| Age of epilepsy onset | -10.7 | 13.0 | 0.8 | 0.42 | -36.9 | 15.6 |
| Male | 4.1 | 492.2 | 0.0 | 0.99 | -993.2 | 1001.5 |
| Number of antiepileptic drugs, n | -38.5 | 63.2 | 0.6 | 0.55 | -166.4 | 89.5 |
| FIQ | -10.6 | 4.1 | 2.6 | 0.15 | -18.9 | 12.2 |
| Absence of cortical lesion on MRI | 0* | . | . | . | . | . |
| Trials beginning with a *wh*-interrogative | -78.7 | 29.9 | 2.6 | 0.01 | -139.3 | -18.1 |

**Supplementary Table S11.** Results of mixed model analysis in the left orbitofrontal region during the 100-ms period immediately after the 2^nd^ phrase offset (period highlighted in the plot above). SE = Standard error, FIQ = Full-scale intelligence quotient, CI = Confidence interval, LL = Lower limit, UL = Upper limit.

* This parameter is set to zero because it is redundant. Compared to trials beginning with a *wh*-interrogative, those beginning with a concrete phrase was associated with 79% greater high gamma activity during the 100-ms period immediately after the 2^nd^ phrase offset.


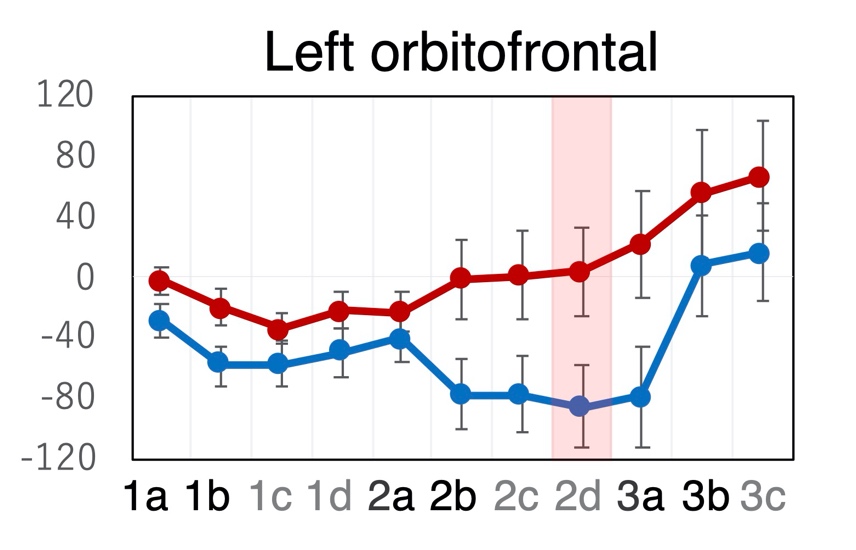


| Parameters | Estimate | SE | \|t\| | Unadjusted *p* | 95%CI LL | 95%CI UL |
| --- | --- | --- | --- | --- | --- | --- |
| Age | 8.6 | 21.7 | 0.4 | 0.69 | -35.3 | 52.6 |
| Age of epilepsy onset | -5.4 | 13.2 | 0.4 | 0.68 | -32.3 | 21.4 |
| Male | -199.1 | 503.7 | 0.4 | 0.70 | -1219.7 | 821.4 |
| Number of antiepileptic drugs, n | -64.7 | 64.6 | 1.0 | 0.32 | -195.7 | 66.2 |
| FIQ | -9.2 | 4.2 | 2.2 | 0.09 | -17.8 | 0.7 |
| Absence of cortical lesion on MRI | 0* | . | . | . | . | . |
| Trials beginning with a *wh*-interrogative | -89.1 | 30.6 | 2.9 | 0.006 | -151.1 | -27.1 |

**Supplementary Table S12.** Results of mixed model analysis in the left orbitofrontal region during the 100-ms period immediately before the 3^rd^ phrase onset (period highlighted in the plot above). SE = Standard error, FIQ = Full-scale intelligence quotient, CI = Confidence interval, LL = Lower limit, UL = Upper limit.

* This parameter is set to zero because it is redundant. Compared to trials beginning with a *wh*-interrogative, those beginning with a concrete phrase was associated with 89% greater high gamma activity during the 100-ms period immediately before the 3^rd^ phrase onset.


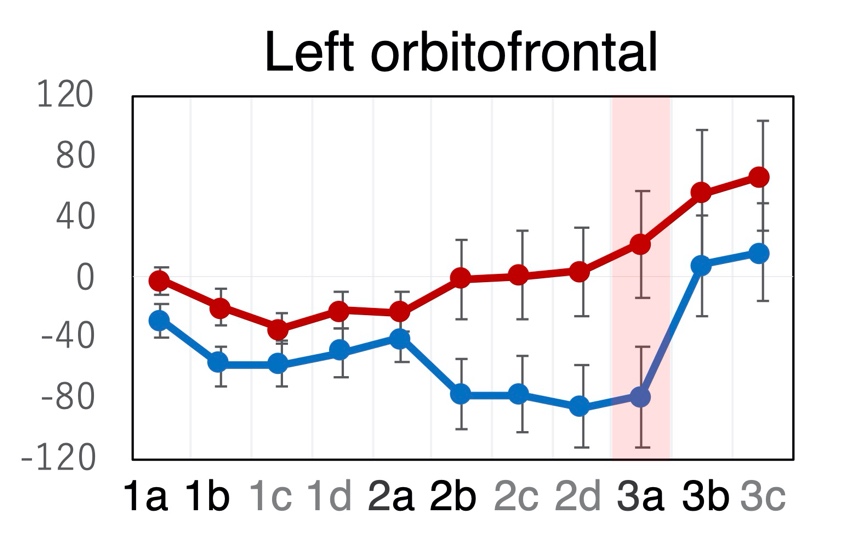


| Parameters | Estimate | SE | \|t\| | Unadjusted *p* | 95%CI LL | 95%CI UL |
| --- | --- | --- | --- | --- | --- | --- |
| Age | 3.2 | 26.3 | 0.1 | 0.90 | -50.1 | 56.6 |
| Age of epilepsy onset | -17.7 | 16.1 | 1.1 | 0.28 | -50.3 | 14.9 |
| Male | -59.1 | 611.6 | 0.1 | 0.92 | -1298.4 | 1180.1 |
| Number of antiepileptic drugs, n | -66.7 | 78.5 | 0.9 | 0.40 | -225.7 | 92.3 |
| FIQ | -14.2 | 5.1 | 2.8 | 0.19 | -24.6 | 32.8 |
| Absence of cortical lesion on MRI | 0* | . | . | . | . | . |
| Trials beginning with a *wh*-interrogative | -100.2 | 37.2 | -2.7 | 0.01 | -175.5 | -24.9 |

**Supplementary Table S13.** Results of mixed model analysis in the left orbitofrontal region during the 100-ms period immediately after the 3^rd^ phrase onset (period highlighted in the plot above). SE = Standard error, FIQ = Full-scale intelligence quotient, CI = Confidence interval, LL = Lower limit, UL = Upper limit.

* This parameter is set to zero because it is redundant. Compared to trials beginning with a *wh*-interrogative, those beginning with a concrete phrase was associated with 100% greater high gamma activity during the 100-ms period immediately after the 3^rd^ phrase onset.


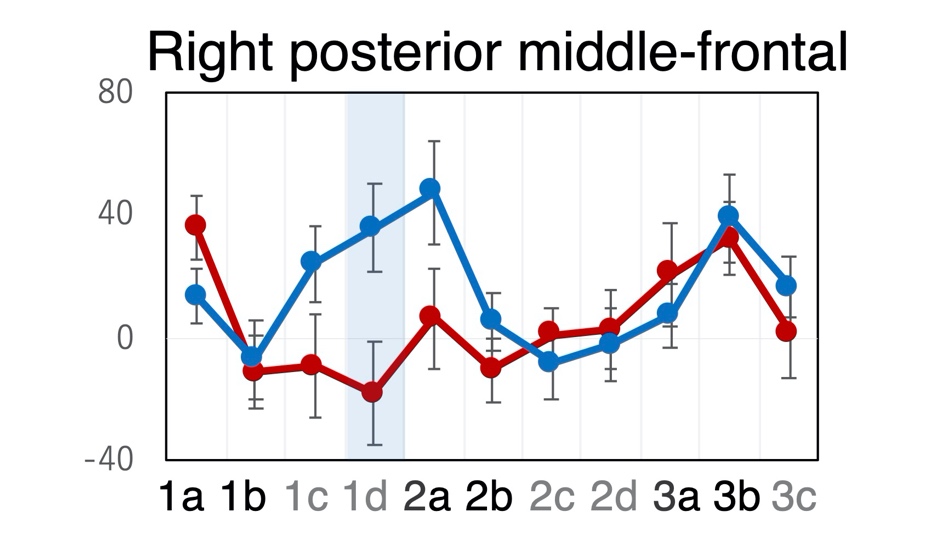


| Parameters | Estimate | SE | \|t\| | Unadjusted *p* | 95%CI LL | 95%CI UL |
| --- | --- | --- | --- | --- | --- | --- |
| Age | -0.8 | 1.3 | 0.7 | 0.52 | -3.4 | 1.7 |
| Age of epilepsy onset | -2.7 | 1.7 | 1.8 | 0.65 | -9.2 | 2.2 |
| Male | 33.6 | 42.9 | 0.5 | 0.10 | -116.8 | 312.4 |
| Number of antiepileptic drugs, n | 0* | . | . | . | . | . |
| FIQ | 0* | . | . | . | . | . |
| Absence of cortical lesion on MRI | 0* | . | . | . | . | . |
| Trials beginning with a *wh*-interrogative | 54.0 | 16.1 | 3.4 | 0.002 | 21.2 | 86.7 |

**Supplementary Table S14.** Results of mixed model analysis in the right posterior middle-frontal gyrus during the 100-ms period immediately before the 2^nd^ phrase onset (period highlighted in the plot above). SE = Standard error, FIQ = Full-scale intelligence quotient, CI = Confidence interval, LL = Lower limit, UL = Upper limit.

* This parameter is set to zero because it is redundant. Compared to trials beginning with a concrete phrase, those beginning with a *wh*-interrogative phrase was associated with 54% greater high gamma activity during the 100-ms period immediately before the 2^nd^ phrase onset.


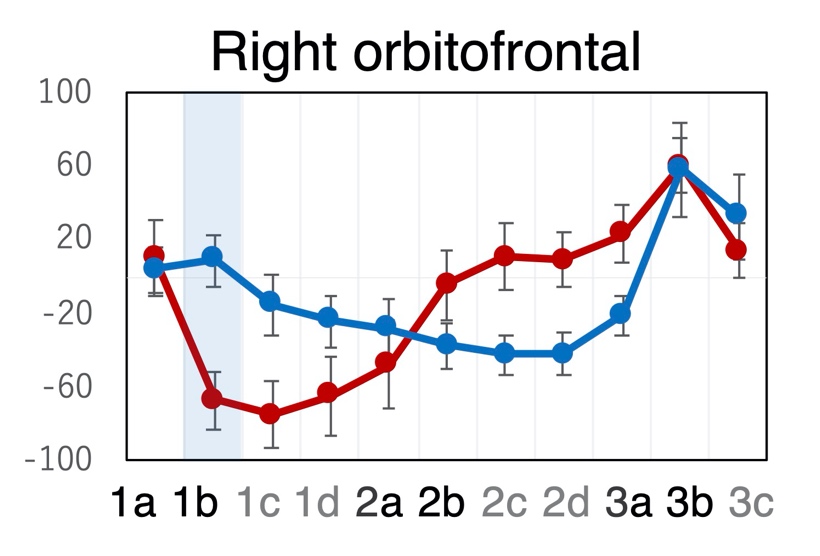


| Parameters | Estimate | SE | \|t\| | Unadjusted *p* | 95%CI LL | 95%CI UL |
| --- | --- | --- | --- | --- | --- | --- |
| Age | -0.2 | 1.4 | 0.1 | 0.90 | -3.0 | 2.7 |
| Age of epilepsy onset | -4.2 | 2.4 | 1.7 | 0.10 | -9.1 | 0.8 |
| Male | 132.3 | 46.9 | 2.8 | 0.11 | -36.6 | 227.9 |
| Number of antiepileptic drugs, n | 0* | . | . | . | . | . |
| FIQ | 0* | . | . | . | . | . |
| Absence of cortical lesion on MRI | 0* | . | . | . | . | . |
| Trials beginning with a *wh*-interrogative | 76.4 | 19.4 | 3.9 | 0.0004 | 36.9 | 115.9 |

**Supplementary Table S15.** Results of mixed model analysis in the right orbitofrontal region during the 100-ms period immediately before the 1^st^ phrase offset (period highlighted in the plot above). SE = Standard error, FIQ = Full-scale intelligence quotient, CI = Confidence interval, LL = Lower limit, UL = Upper limit.

* This parameter is set to zero because it is redundant. Compared to trials beginning with a concrete phrase, those beginning with a *wh*-interrogative phrase was associated with 76% greater high gamma activity during the 100-ms period immediately before the 1^st^ phrase offset.

| Type-A question list | Corresponding Type-B question | 1st phrase | 1st phrase  (English translation) | 2nd phrase | 2nd phrase  (English translation) | 3rd phrase | 3rd phrase  (English translation) | |
| --- | --- | --- | --- | --- | --- | --- | --- | --- |
| QA1 | QB56 | どこで | where | ほんを | books | かりるの | borrow |  |
| QA2 | QB29 | りんごを | apples | かうのは | buy | どこ | where |  |
| QA3 | QB46 | こいのぼりを | carp streamers | あげるのは | hang up | いつ | when |  |
| QA4 | QB30 | いつ | when | おせちりょうりを | new year's dishes | たべるの | eat |  |
| QA5 | QB80 | どこで | where | ボートを | a boat | こぐの | row |  |
| QA6 | QB68 | なにが | what | かじを | fire | けすの | put out |  |
| QA7 | QB59 | なにが | what | みつを | nectar | すうの | suck |  |
| QA8 | QB25 | ゴリラを | gorillas | みるのは | see | どこ | where |  |
| QA9 | QB87 | ようふくを | clothes | あらうのは | wash | なに | what |  |
| QA10 | QB33 | ささを | bamboos | たべるのは | eat | なに | what |  |
| QA11 | QB85 | やまを | mountains | のぼるのは | climb | いつ | when |  |
| QA12 | QB96 | ジュースを | juice | ひやすのは | cool | どこ | where |  |
| QA13 | QB67 | はを | teeth | みがくのは | brush | いつ | when |  |
| QA14 | QB32 | なにが | what | さばくを | in the desert | あるくの | walk |  |
| QA15 | QB27 | いつ | when | うみを | in the sea | およぐの | swim |  |
| QA16 | QB55 | おかしを | sweets | たべるのは | eat | いつ | when |  |
| QA17 | QB58 | どこで | where | ゆうしょくを | dinner | たべるの | eat |  |
| QA18 | QB11 | よごれを | dirt | おとすのは | remove | なに | what |  |
| QA19 | QB36 | なにが | what | てがみを | mails | あつめるの | collect |  |
| QA20 | QB69 | なにが | what | すを | nests | つくるの | build |  |
| QA21 | QB84 | つきを | moon | みるのは | see | いつ | when |  |
| QA22 | QB91 | きゃくを | customers | のせるのは | pick up | なに | what |  |
| QA23 | QB48 | いつ | when | ケーキを | cakes | たべるの | eat |  |
| QA24 | QB52 | いつ | when | しんぶんを | newspapers | よむの | read |  |
| QA25 | QB57 | にんじんを | carrots | たべるのは | eat | なに | what |  |
| QA26 | QB62 | なにが | what | じめんを | on the ground | はうの | crawl |  |
| QA27 | QB54 | なにが | what | そうげんを | on the meadow | はしるの | run |  |
| QA28 | QB26 | ねんがじょうを | new-year's-cards | おくるのは | send | いつ | when |  |
| QA29 | QB66 | プレゼントを | gifts | とどけるのは | deliver | なに | what |  |
| QA30 | QB41 | どこで | where | さらを | dishes | あらうの | wash |  |
| QA31 | QB76 | いつ | when | すいかを | sweet melons | たべるの | eat |  |
| QA32 | QB18 | いつ | when | コートを | coats | きるの | wear |  |
| QA33 | QB92 | おふろを | baths | わかすのは | boil | いつ | when |  |
| QA34 | QB16 | いつ | when | パジャマを | pajamas | きるの | wear |  |
| QA35 | QB75 | どうろを | on the road | はしるのは | run | なに | what |  |
| QA36 | QB1 | いつ | when | みそしるを | miso-soup | のむの | drink |  |
| QA37 | QB93 | おとを | sounds | だすのは | make | なに | what |  |
| QA38 | QB90 | みずぎを | swimsuit | きるのは | wear | いつ | when |  |
| QA39 | QB34 | ハンバーグを | hamburg steak | たべるのは | eat | どこ | where |  |
| QA40 | QB50 | ふとんを | futons | たたむのは | fold | いつ | when |  |
| QA41 | QB64 | なにが | what | そらを | in the sky | とぶの | fly |  |
| QA42 | QB70 | じどうしゃを | cars | つくるのは | make | どこ | where |  |
| QA43 | QB86 | かみのけを | hair | きるのは | cut | どこ | where |  |
| QA44 | QB45 | なにが | what | うみを | in the sea | およぐの | swim |  |
| QA45 | QB40 | どこで | where | ひまわりを | sunflowers | うえるの | plant |  |
| QA46 | QB24 | どこで | where | チャーハンを | fried rice | たべるの | eat |  |
| QA47 | QB43 | ごみを | garbage | すうのは | suck up | なに | what |  |
| QA48 | QB71 | なにが | what | にもつを | baggage | はこぶの | carry |  |
| QA49 | QB21 | おかねを | money | あずけるのは | deposit | どこ | where |  |
| QA50 | QB13 | せんぷうきを | fans | まわすのは | turn on | いつ | when |  |
| QA51 | QB81 | てぶくろを | gloves | はめるのは | put on | いつ | when |  |
| QA52 | QB95 | ピアノを | piano | ひくのは | play | どこ | where |  |
| QA53 | QB63 | てを | hands | あらうのは | wash | どこ | where |  |
| QA54 | QB94 | なにが | what | くさを | grass | たべるの | eat |  |
| QA55 | QB15 | どこで | where | むしを | insects | つかまえるの | catch |  |
| QA56 | QB3 | どこで | where | えを | pictures | かくの | draw |  |
| QA57 | QB82 | なにが | what | たまごを | eggs | うむの | spawn |  |
| QA58 | QB89 | きを | trees | のぼるのは | climb | なに | what |  |
| QA59 | QB4 | なにが | what | バナナを | bananas | たべるの | eat |  |
| QA60 | QB61 | なにが | what | ネズミを | rats | おうの | chase |  |
| QA61 | QB10 | もちを | mochi (pound steamed rice) | つくのは | pound | いつ | when |  |
| QA62 | QB19 | スキーを | ski | すべるのは | skiing | どこ | where |  |
| QA63 | QB74 | いつ | when | おちばを | fallen leaves | ひろうの | glean |  |
| QA64 | QB8 | たこを | kites | あげるのは | fly | どこ | where |  |
| QA65 | QB20 | どこで | where | てがみを | mails | おくるの | send |  |
| QA66 | QB5 | どんぐりを | acorns | あつめるのは | collect | なに | what |  |
| QA67 | QB17 | どこで | where | やさいを | vegetables | きるの | cut |  |
| QA68 | QB14 | いつ | when | シャワーを | a shower | あびるの | take |  |
| QA69 | QB77 | どこで | where | えいごを | English | ならうの | learn |  |
| QA70 | QB51 | どこで | where | チョコレートを | chocolates | かうの | buy |  |
| QA71 | QB6 | いつ | when | うんどうかいを | a sport-day | やるの | join |  |
| QA72 | QB73 | ストーブを | stoves | つけるのは | turn on | いつ | when |  |
| QA73 | QB88 | いつ | when | ほしを | stars | みるの | see |  |
| QA74 | QB44 | シャワーを | a shower | あびるのは | take | どこ | where |  |
| QA75 | QB2 | はなびを | fireworks | あげるのは | launch | いつ | when |  |
| QA76 | QB38 | ごはんを | food | あたためるのは | heat | なに | what |  |
| QA77 | QB23 | おゆを | hot water | わかすのは | boil | なに | what |  |
| QA78 | QB78 | シャツを | shirts | しまうのは | put away | どこ | where |  |
| QA79 | QB9 | どこで | where | くつを | shoes | はくの | put on |  |
| QA80 | QB12 | いつ | when | まめを | beans | まくの | sowing |  |
| QA81 | QB31 | どこで | where | そりを | sleds | すべるの | sledding |  |
| QA82 | QB7 | どこで | where | きんぎょを | goldfishes | そだてるの | grow |  |
| QA83 | QB79 | かさを | umbrellas | つかうのは | use | いつ | when |  |
| QA84 | QB42 | いつ | when | プレゼントを | gifts | もらうの | get |  |
| QA85 | QB83 | どこで | where | きりんを | giraffes | みるの | see |  |
| QA86 | QB28 | びょうきを | disease | なおすのは | cure | どこ | where |  |
| QA87 | QB39 | なにが | what | あなを | dig | ほるの | holes |  |
| QA88 | QB47 | さんすうを | math | まなぶのは | learn | どこ | where |  |
| QA89 | QB53 | しゃしんを | pictures | とるのは | take | なに | what |  |
| QA90 | QB37 | もじを | sentence | けすのは | erase | なに | what |  |
| QA91 | QB35 | なにが | what | しっぽを | tails | ふるの | wag |  |
| QA92 | QB60 | かきごおりを | shaved ice | たべるのは | eat | いつ | when |  |
| QA93 | QB65 | いつ | when | おひなさまを | Japanese Hina dolls | かざるの | display |  |
| QA94 | QB72 | かわを | in the river | およぐのは | swim | なに | what |  |
| QA95 | QB22 | いつ | when | むぎちゃを | barley tea | のむの | drink |  |
| QA96 | QB49 | あにめを | anime | みるのは | watch | どこ | where |  |

**Supplementary Table S16.** List of Type-A questions. Patients #1, #2, #3, #7, #10, #12, #14, #15, #17, #19, and #20 were assigned Type-A questions.

| Type-B question list | Corresponding Type-A question | 1st phrase | 1st phrase  (English translation) | 2nd phrase | 2nd phrase  (English translation) | 3rd phrase | 3rd phrase  (English translation) | |
| --- | --- | --- | --- | --- | --- | --- | --- | --- |
| QB1 | QA36 | みそしるを | miso-soup | のむのは | drink | いつ | when |  |
| QB2 | QA75 | いつ | when | はなびを | fireworks | あげるの | launch |  |
| QB3 | QA56 | えを | pictures | かくのは | draw | どこ | where |  |
| QB4 | QA59 | バナナを | bananas | たべるのは | eat | なに | what |  |
| QB5 | QA66 | なにが | what | どんぐりを | acorns | あつめるの | collect |  |
| QB6 | QA71 | うんどうかいを | a sport-day | やるのは | join | いつ | when |  |
| QB7 | QA82 | きんぎょを | goldfishes | そだてるのは | grow | どこ | where |  |
| QB8 | QA64 | どこで | where | たこを | kites | あげるの | fly |  |
| QB9 | QA79 | くつを | shoes | はくのは | put on | どこ | where |  |
| QB10 | QA61 | いつ | when | もちを | mochi (pound steamed rice) | つくの | pound |  |
| QB11 | QA18 | なにが | what | よごれを | dirt | おとすの | removes |  |
| QB12 | QA80 | まめを | beans | まくのは | sowing | いつ | when |  |
| QB13 | QA50 | いつ | when | せんぷうきを | fans | まわすの | turn on |  |
| QB14 | QA68 | シャワーを | a shower | あびるのは | take | いつ | when |  |
| QB15 | QA55 | むしを | insects | つかまえるのは | catch | どこ | where |  |
| QB16 | QA34 | パジャマを | pajamas | きるのは | wear | いつ | when |  |
| QB17 | QA67 | やさいを | vegetables | きるのは | cut | どこ | where |  |
| QB18 | QA32 | コートを | coats | きるのは | wear | いつ | when |  |
| QB19 | QA62 | どこで | where | スキーを | ski | すべるの | skiing |  |
| QB20 | QA65 | てがみを | mails | おくるのは | send | どこ | where |  |
| QB21 | QA49 | どこで | where | おかねを | money | あずけるの | deposit |  |
| QB22 | QA95 | むぎちゃを | barley tea | のむのは | drink | いつ | when |  |
| QB23 | QA77 | なにが | what | おゆを | hot water | わかすの | boil |  |
| QB24 | QA46 | チャーハンを | fried rice | たべるのは | eat | どこ | where |  |
| QB25 | QA8 | どこで | where | ゴリラを | gorillas | みるの | see |  |
| QB26 | QA28 | いつ | when | ねんがじょうを | new-year's-cards | おくるの | send |  |
| QB27 | QA15 | うみを | in the sea | およぐのは | swim | いつ | when |  |
| QB28 | QA86 | どこで | where | びょうきを | disease | なおすの | cure |  |
| QB29 | QA2 | どこで | where | りんごを | apples | かうの | buy |  |
| QB30 | QA4 | おせちりょうりを | new year's dishes | たべるのは | eat | いつ | when |  |
| QB31 | QA81 | そりを | sleds | すべるのは | sledding | どこ | where |  |
| QB32 | QA14 | さばくを | in the desert | あるくのは | walk | なに | what |  |
| QB33 | QA10 | なにが | what | ささを | bamboos | たべるの | eat |  |
| QB34 | QA39 | どこで | where | ハンバーグを | hamburg steak | たべるの | eat |  |
| QB35 | QA91 | しっぽを | tails | ふるのは | wag | なに | what |  |
| QB36 | QA19 | てがみを | mails | あつめるのは | collect | なに | what |  |
| QB37 | QA90 | なにが | what | もじを | sentence | けすの | erase |  |
| QB38 | QA76 | なにが | what | ごはんを | food | あたためるの | heat |  |
| QB39 | QA87 | あなを | holes | ほるのは | dig | なに | what |  |
| QB40 | QA45 | ひまわりを | sunflowers | うえるのは | plant | どこ | where |  |
| QB41 | QA30 | さらを | dishes | あらうのは | wash | どこ | where |  |
| QB42 | QA84 | プレゼントを | gifts | もらうのは | get | いつ | when |  |
| QB43 | QA47 | なにが | what | ごみを | garbage | すうの | suck up |  |
| QB44 | QA74 | どこで | where | シャワーを | shower | あびるの | take |  |
| QB45 | QA44 | うみを | in the sea | およぐのは | swim | なに | what |  |
| QB46 | QA3 | いつ | when | こいのぼりを | carp streamers | あげるの | hang up |  |
| QB47 | QA88 | どこで | where | さんすうを | math | まなぶの | learn |  |
| QB48 | QA23 | ケーキを | cake | たべるのは | eat | いつ | when |  |
| QB49 | QA96 | どこで | where | アニメを | anime | みるの | watch |  |
| QB50 | QA40 | いつ | when | ふとんを | futon | たたむの | fold |  |
| QB51 | QA70 | チョコレートを | chocolates | かうのは | buy | どこ | where |  |
| QB52 | QA24 | しんぶんを | newspapers | よむのは | read | いつ | when |  |
| QB53 | QA89 | なにが | what | しゃしんを | pictures | とるの | take |  |
| QB54 | QA27 | そうげんを | on the meadow | はしるのは | run | なに | what |  |
| QB55 | QA16 | いつ | when | おかしを | sweets | たべるの | eat |  |
| QB56 | QA1 | ほんを | books | かりるのは | borrow | どこ | where |  |
| QB57 | QA25 | なにが | what | にんじんを | carrots | たべるの | eat |  |
| QB58 | QA17 | ゆうしょくを | dinner | たべるのは | eat | どこ | where |  |
| QB59 | QA7 | みつを | nectar | すうのは | suck | なに | what |  |
| QB60 | QA92 | いつ | when | かきごおりを | shaved ice | たべるの | eat |  |
| QB61 | QA60 | ねずみを | rats | おうのは | chase | なに | what |  |
| QB62 | QA26 | じめんを | on the ground | はうのは | crawl | なに | what |  |
| QB63 | QA53 | どこで | where | てを | hands | あらうの | wash |  |
| QB64 | QA41 | そらを | in the sky | とぶのは | fly | なに | what |  |
| QB65 | QA93 | おひなさまを | Japanese Hina dolls | かざるのは | display | いつ | when |  |
| QB66 | QA29 | なにが | what | プレゼントを | gifts | とどけるの | deliver |  |
| QB67 | QA13 | いつ | when | はを | teeth | みがくの | brush |  |
| QB68 | QA6 | かじを | fire | けすのは | put out | なに | what |  |
| QB69 | QA20 | すを | nest | つくるのは | build | なに | what |  |
| QB70 | QA42 | どこで | where | じどうしゃを | car | つくるの | make |  |
| QB71 | QA48 | にもつを | baggage | はこぶのは | carry | なに | what |  |
| QB72 | QA94 | なにが | what | かわを | in the river | およぐの | swim |  |
| QB73 | QA72 | いつ | when | ストーブを | stoves | つけるの | turn on |  |
| QB74 | QA63 | おちばを | fallen leaves | ひろうのは | glean | いつ | when |  |
| QB75 | QA35 | なにが | what | どうろを | on the road | はしるの | run |  |
| QB76 | QA31 | すいかを | sweet melons | たべるのは | eat | いつ | when |  |
| QB77 | QA69 | えいごを | English | ならうのは | learn | どこ | where |  |
| QB78 | QA78 | どこで | where | シャツを | shirts | しまうの | put away |  |
| QB79 | QA83 | いつ | when | かさを | umbrellas | つかうの | use |  |
| QB80 | QA5 | ボートを | boats | こぐのは | row | どこ | where |  |
| QB81 | QA51 | いつ | when | てぶくろを | gloves | はめるの | put on |  |
| QB82 | QA57 | たまごを | eggs | うむのは | spawn | なに | what |  |
| QB83 | QA85 | きりんを | giraffes | みるのは | see | どこ | where |  |
| QB84 | QA21 | いつ | when | つきを | moon | みるの | see |  |
| QB85 | QA11 | いつ | when | やまを | mountains | のぼるの | climb |  |
| QB86 | QA43 | どこで | where | かみのけを | hair | きるの | cut |  |
| QB87 | QA28 | なにが | what | ようふくを | clothes | あらうの | wash |  |
| QB88 | QA73 | ほしを | stars | みるのは | see | いつ | when |  |
| QB89 | QA58 | なにが | what | きを | trees | のぼるの | climb |  |
| QB90 | QA38 | いつ | when | みずぎを | swimsuits | きるの | wear |  |
| QB91 | QA22 | なにが | what | きゃくを | customers | のせるの | pick up |  |
| QB92 | QA33 | いつ | when | おふろを | baths | わかすの | boil |  |
| QB93 | QA37 | なにが | what | おとを | a sound | だすの | make |  |
| QB94 | QA54 | くさを | grasses | たべるのは | eat | なに | what |  |
| QB95 | QA52 | どこで | where | ピアノを | pianos | ひくの | play |  |
| QB96 | QA12 | どこで | where | ジュースを | juice | ひやすの | cool |  |

**Supplementary Table S17.** List of Type-B questions. Patients #4, #5, #6, #8, #9, #11, #13, #16, #18, #21, #22, and #23 were assigned Type-B questions.
